# The utility of a genetic kidney disease clinic employing a broad range of genomic testing platforms: experience of the Irish Kidney Gene Project

**DOI:** 10.1007/s40620-021-01236-2

**Published:** 2022-01-31

**Authors:** Elhussein A. E. Elhassan, Susan L. Murray, Dervla M. Connaughton, Claire Kennedy, Sarah Cormican, Cliona Cowhig, Caragh Stapleton, Mark A. Little, Kendrah Kidd, Anthony J. Bleyer, Martina Živná, Stanislav Kmoch, Neil K. Fennelly, Brendan Doyle, Anthony Dorman, Matthew D. Griffin, Liam Casserly, Peter C. Harris, Friedhelm Hildebrandt, Gianpiero L. Cavalleri, Katherine A. Benson, Peter J. Conlon

**Affiliations:** 1 Department of Nephrology and Transplantation, Beaumont Hospital, Dublin, Ireland; 2 Department of Medicine, Dublin, Royal College of Surgeons in Ireland, Dublin, Ireland; 3 Schulich School of Medicine and Dentistry, University of Western Ontario, London, ON Canada; 4 Division of Nephrology, Department of Medicine, London Health Sciences Centre, London, ON Canada; 5 School of Pharmacy and Biomolecular Sciences, Royal College of Surgeons, Dublin, Ireland; 6 Trinity Health Kidney Centre, Trinity Translational Medicine Institute, Trinity College Dublin, St James’ Street, Dublin 8, Ireland; 7 Section on Nephrology, Wake Forest School of Medicine, Winston-Salem, NC USA; 8 Research Unit for Rare Diseases, Department of Paediatrics and Inherited Metabolic Disorders, First Faculty of Medicine, Charles University in Prague, Prague, Czech Republic; 9 Department of Pathology, Beaumont Hospital, Dublin, Ireland; 10 Department of Pathology, Royal College of Surgeons in Ireland, Dublin, Ireland; 11 Nephrology Department, Galway University Hospitals, Saolta University Healthcare Group, Galway, Ireland; 12 Regenerative Medicine Institute (REMEDI) at CÚRAM Centre for Research in Medical Devices, School of Medicine, National University of Ireland, Galway, Ireland; 13 Department of Nephrology and Internal Medicine, University Hospital Limerick, Limerick, Ireland; 14 Division of Nephrology and Hypertension, Mayo Clinic, Rochester, MN USA; 15 Department of Paediatrics, Boston Children’s Hospital, Harvard Medical School, Boston, MA 02115 USA

**Keywords:** Chronic kidney disease, Inherited kidney diseases, Next-generation sequencing, Polycystic kidney genetics, Genetic kidney disease

## Abstract

**Background and aims:**

Genetic testing presents a unique opportunity for diagnosis and management of genetic kidney diseases (GKD). Here, we describe the clinical utility and valuable impact of a specialized GKD clinic, which uses a variety of genomic sequencing strategies.

**Methods:**

In this prospective cohort study, we undertook genetic testing in adults with suspected GKD according to prespecified criteria. Over 7 years, patients were referred from tertiary centres across Ireland to an academic medical centre as part of the Irish Kidney Gene Project.

**Results:**

Among 677 patients, the mean age was of 37.2 ± 13 years, and 73.9% of the patients had family history of chronic kidney disease (CKD). We achieved a molecular diagnostic rate of 50.9%. Four genes accounted for more than 70% of identified pathogenic variants: *PKD1* and *PKD2* (*n* = 186, 53.4%), *MUC1* (8.9%), and *COL4A5* (8.3%). In 162 patients with a genetic diagnosis, excluding *PKD1*/*PKD2*, the a priori diagnosis was confirmed in 58% and in 13% the diagnosis was reclassified. A genetic diagnosis was established in 22 (29.7%) patients with CKD of uncertain aetiology. Based on genetic testing, a diagnostic kidney biopsy was unnecessary in 13 (8%) patients. Presence of family history of CKD and the underlying a priori diagnosis were independent predictors (*P* < 0.001) of a positive genetic diagnosis.

**Conclusions:**

A dedicated GKD clinic is a valuable resource, and its implementation of various genomic strategies has resulted in a direct, demonstrable clinical and therapeutic benefits to affected patients.

**Graphical abstract:**

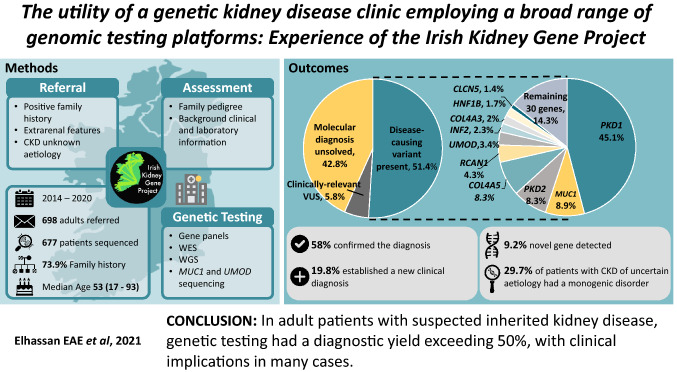

**Supplementary Information:**

The online version contains supplementary material available at 10.1007/s40620-021-01236-2.

## Introduction

Testing for genetic kidney diseases (GKD), encompasses an array of more than 150 rare monogenic disorders, uncovers new horizons for diagnosis and management of patients and their families [1, 2]. Up to 35% of adults with chronic kidney disease (CKD) report a positive family history, suggesting a hereditary element [3]. While a strong genetic component of certain forms of GKD such as autosomal dominant polycystic kidney disease (ADPKD) is well recognised, other forms of adult GKD have historically been overlooked, and equally can be complex and multifaceted [4, 5].

The establishment of a specialised genetics service, utilising a multidisciplinary team (MDT) of clinical nephrologists, clinical geneticists, genetics counsellors, nurses, pathologists, and research geneticists/bioinformaticians is warranted to diagnose, manage, and treat patients with GKD [6]. Therefore, to determine the efficacy of genomic sequencing technologies, including next-generation sequencing (NGS), and the underlying genetic cause of disease in patients with suspected GKD, we established a research program known as the Irish Kidney Gene Project (IKGP) and an associated clinical service called the Genetic Kidney Disease Clinic (GKDC), at Beaumont Hospital, Dublin, Ireland [7, 8].

To diagnose GKD, several approaches can be adopted. Recent studies have focused on the utility of whole-exome sequencing (WES) in diagnosing GKD, with a diagnostic yield of 9–37% for monogenic disease depending on the patient population [9–12]. While WES is undoubtedly useful, it may not be practical or cost-efficient for all GKD clinics. Indeed, WES has been described as ineffective for diagnosis of ADPKD, the most common form of GKD [13]. NGS-based targeted gene panels may be considered as an alternative, with several studies reporting diagnostic rates ranging from 20 to 78% [14–16]. Other techniques may be required for specific genetic diseases. For example, the *MUC1* gene contains a highly repetitive region with a high guanosine/cytosine content, resulting in the inability of WES and NGS panels to identify *MUC1* variants, one of the most common causes of autosomal dominant tubulointerstitial kidney disease (ADTKD) [17]. Specialized testing is required for this condition [18]. Finally, the use of WES or indeed whole genome sequencing (WGS) allows future-proofing of diagnostic tests, allowing for reanalysis of data as novel genes are discovered. Through integration with research centres, WES and WGS can be utilised to assist in the identification of novel GKD genes. Thus, a multi-faceted approach provides the best opportunity to supply genetic diagnoses.

In this prospective study, we describe our overall experience of the IKGP over the 7-year period from 2014 to 2020 and the clinical impact of GKDC on patients, including some patient subsets that have been previously described [11, 19–24].

## Methods

### Patient data

Adult patients attending a university-based academic Department of Nephrology, the Irish National Kidney Transplant Centre, at Beaumont Hospital, Dublin were recruited into this prospective cohort study. Ethical approval was sought and granted by the Ethics Review Board of Beaumont Hospital (REC 19/28). All patients gave explicit informed consent to participate.

Letters were sent out to nephrologists nationwide to inform them of the service and invite them to refer any adult patient (age ≥ 18 years) with CKD who had either a positive family history, extrarenal features, or had CKD of ‘’uncertain aetiology’’ (uCKD). Depending on the clinical and histological findings of the nephrologists’ referrals, patients were grouped into seven categories of a priori clinical diagnoses. A detailed description of these categories and the diagnostic genomic methods used are listed in the Supplementary Material.

All patients referred to the GKDC, were reviewed and counselled by two among the following trained nephrologists—PC, CK, DC, SM, EE—with an interest in GKD and underwent research genetic testing guided by the a priori diagnosis.

### Genetic diagnosis

The choice of genetic testing was guided by the patient’s a priori diagnosis, the likely success of sequencing strategies and cost considerations*.* Sequencing and bioinformatics analyses for gene panels [19, 23], WES [11, 22, 25] and WGS [24] were performed by DMC, PCH, FH, GLC, KAB as described previously(see Supplementary Material). *MUC1* genotyping [18] was undertaken at the Broad Institute, while immunostaining for MUC1fs in urinary cell smears or kidney biopsy [20] followed by entire *MUC1* sequencing using either Illumina [20] or PacBio Single Molecule, Real-Time (SMRT) Sequencing [26] were provided by Charles University in Prague by KK, AJB, MZ, SK. In each case these results of genomic testing were assessed by the MDT of clinical nephrologists with specific experience and training in GKD together with experts in clinical genetics and bioinformatics. Variants were prioritised and classified as per the American College of Medical Genetics (ACMG) guidelines [27].

Where a genetic diagnosis was made, patients were invited by the clinic to undergo a confirmatory genetic testing at an accredited clinical lab using a second sample, along with counselling on the implications of the results on their management and on other family members. This was required to ensure correct governance when including the test result in the clinical record and ensured complete accuracy of both the variant identification and interpretation. Only ACMG likely pathogenic/pathogenic results replicated at the clinical lab were returned to patients.

### Statistics

Patient characteristics and genetic diagnosis were collected, and descriptive statistics were expressed as mean ± SD, percentages or median [interquartile range, IQR]. We evaluated clinical predictors favouring identification of a genetic diagnosis using logistic regression analyses. Data were analysed using STATA SE (version 16 StataCorp, College Station, TX, USA). Probability of a type 1 error less than 0.05 was statistically significant.

## Results

### Cohort description

**Fig. 1:**
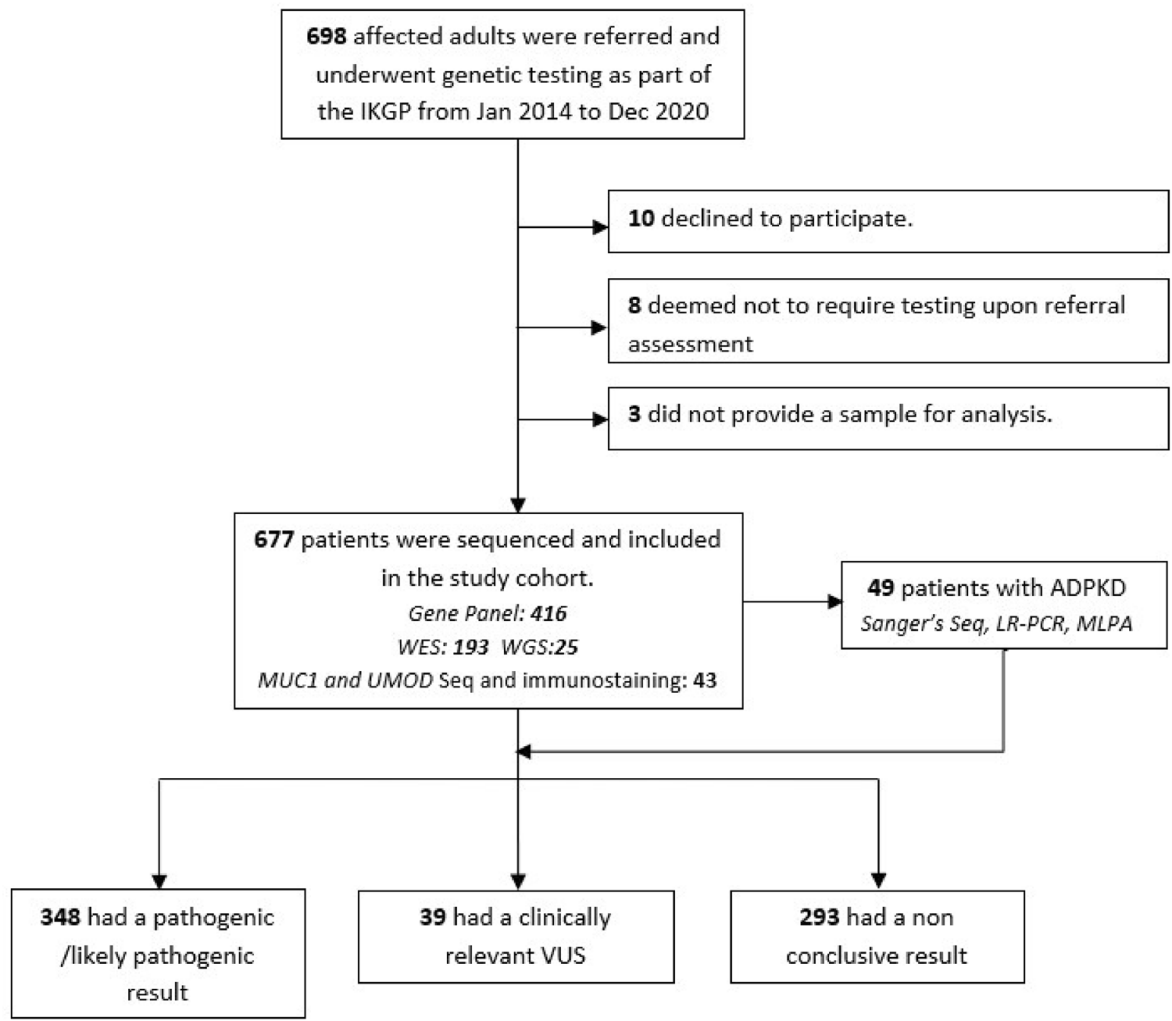
Flowchart of genetic kidney disease clinic recruitment, sequencing technologies, and outcome. *IKGP* Irish Kidney Gene Project, *MUC1* mucin 1 gene, *MLPA* multiplex ligation-dependent probe amplification, *LR-PCR* long-range polymerase chain reaction, *UMOD* Uromodulin, *VUS* variant of uncertain significance, *WES* whole–exome sequencing, *WGS* whole–genome sequencing

A total of 698 affected adult individuals (*n* = 522 families) were referred to the GKD service, none of whom had undergone prior genomic testing. Twenty-one patients were excluded from analysis as they declined participation, were deemed not to require testing upon referral assessment, or did not provide a sample for analysis (Fig. 1). The remaining 677 adults (*n* = 501 families) underwent genetic sequencing and formed the study cohort. Table 1 presents the baseline characteristics of the patients stratified by their genomic sequencing status.

**Table 1:** Characteristics of the 677 affected individuals (501 families) sequenced by the Irish Kidney Gene Project (IKGP)

Characteristics	Total sequenced (*n* = 677)	Total variants identified (*n* = 387)	Unsolved (*n* = 290)	*P* value
A priori clinical diagnosis, *n* (%)				
PKD	241 (35.6)	205 (53)	36 (12.4)	< 0.001
CAKUT	85 (12.6)	12 (3.1)	73 (25.2)	
Chronic GN	112 (16.5)	24 (6.2)	88 (30.3)	
TIKD	75 (11.1)	49 (12.7)	26 (9)	
AS/FSGS	72 (10.6)	57 (14.7)	15 (5.2)	
Others	18 (2.7)	13 (3.3)	5 (1.7)	
uCKD	74 (10.9)	27 (7)	47 (16.2)	
Recruited from, *n* (%)^1^				
Monogenic kidney disease study	138 (20.4)	56 (14.5)	82 (28.3)	< 0.001
PKD study	208 (30.7)	177 (45.7)	31 (10.7)	
GKD clinic	331 (7.8)	154 (39.8)	177 (61)	
Median age, yrs (range)	53 (18–93)	54 (18–88)	51 (18–93)	0.018
Age in years at onset of disease, *n* (%)				
< 18 (childhood onset)	118 (17.4)	66 (17.1)	52 (17.9)	0.142
≥ 18 (adult onset)	450 (66.5)	274 (70.8)	176 (60.7)	
Unavailable	109 (16.1)	47 (12.1)	62 (21.4)	
ESKD, *n* (%)				
Yes	440 (65)	228 (58.9)	212 (73.1)	0.001
No	215 (31.8)	142 (36.7)	73 (25.2)	
Missing	22 (3.2)	17 (4.4)	5 (1.7)	
Median age at onset of ESKD, [IQR]	30 [20–43]	30 [21–44]	30 [18–43]	0.425
Sex				
Male	358 (52.9)	196 (50.6)	162 (55.8)	0.163
Female	319 (47.1)	191 (49.4)	128 (44.2)	
FHx of CKD, *n* (%) ^2^				
Yes	500 (73.9)	336 (86.8)	164 (56.6)	< 0.001
No	140 (20.7)	43 (11.1)	97 (33.4)	
Unavailable	37 (5.4)	8 (2.1)	29 (10)	
Self-reported ethnicity				
Irish	646 (95.4)	373 (96.4)	273 (94.2)	0.551
Other Europeans	18 (2.7)	8 (2.1)	10 (3.4)	
Black	8 (1.2)	4 (1)	4 (1.4)	
Asian	5 (0.7)	2 (0.5)	3 (1)	

*AS* Alport syndrome, *CAKUT* congenital anomalies of the kidney and urinary tract, *CKD* Chronic kidney disease, *ESKD* end stage kidney disease, *GKD* genetic kidney disease, *IQR* Interquartile range, *FHx* family history, *FSGS* focal segmental glomerulosclerosis, *GN* glomerulonephritis, *PKD* polycystic kidney disease, *uCKD* CKD of uncertain aetiology, *TIKD* tubulointerstitial kidney disease, *Yrs* years

^1^In total, 677 patients were reviewed in the genetic kidney disease (GKD) clinic, recruited and bio-banked for the evaluation and management of nephropathy. Included in this large cohort, two groups of patients were previously published in the Monogenic Kidney Disease Study [11] and PKD study [23]; these are grouped separately for clarity

^2^A positive family history of kidney disease that was reported by the patient (either a 1st-degree relative (parent, child, or sibling) or a 2nd -degree relative (grandparent, aunt, uncle, niece, nephew, or cousin)

The study population had a slight male preponderance, with 358 (52.9%) males. One-hundred and eighteen (17.4%) patients had disease onset at < 18 years of age with a mean age of 10.2 ± 5.7 years, and 450 (66.5%) patients presented as adults, with a mean age of 37.2 ± 13 years. Five hundred (73.9%) participants reported a family history of renal disease (*P* =  < 0.001). Sixty-five% of patients reached end stage kidney disease (ESKD) at the last review, with a median age at ESKD of 30 (interquartile range (IQR) 20–43) years. Two hundred fifteen (31.8%) patients had CKD, defined as decreased estimated glomerular filtration rate lower than 60 ml/min/1.73 m^2^ for 3 months or longer, by 47 (IQR 37–59) years of age. A total of 664 (98%) were self-reported as Caucasian, and more than 95% of the cohort were Irish, which is representative of the Irish population [28]. None of the patients reported consanguinity.

**Fig. 2:**
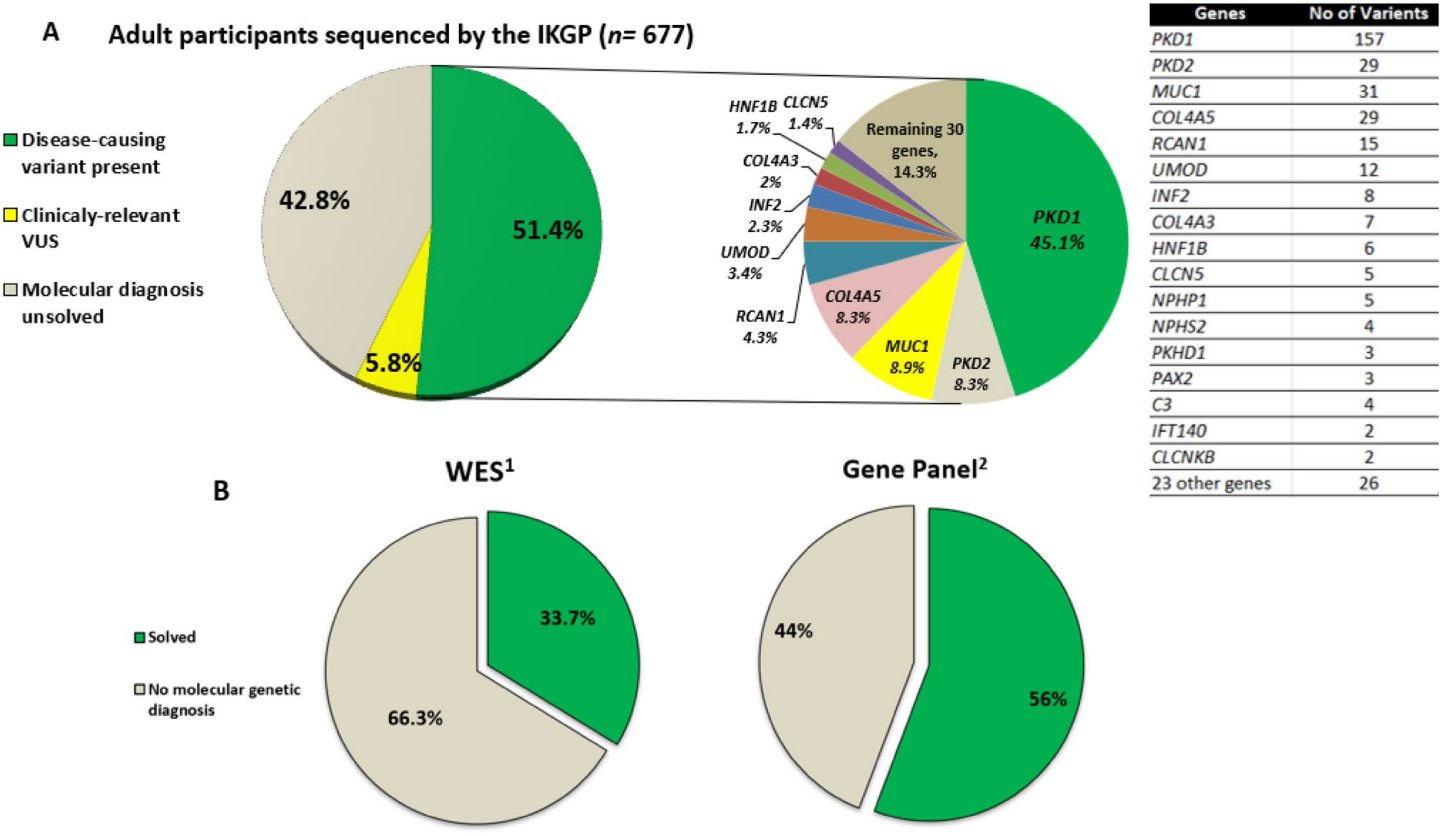
**a** Breakdown of disease-causing genes containing a pathogenic variant detected by the Irish Kidney Gene Project in whole cohort. **b** Pathogenic detection rate in whole-exome sequencing (WES) and a targeted gene panel. *IKGP* Irish Kidney Gene Project, *VUS* variant of unknown significance. ^1^More than 475 known chronic kidney disease genes; see references [11, 25]. ^2^Roche SeqCap EZ Choice (227 genes panel) and Roche NimbleGen HeatSeq panel (11 genes panel); see reference [23]

**Fig. 3:**
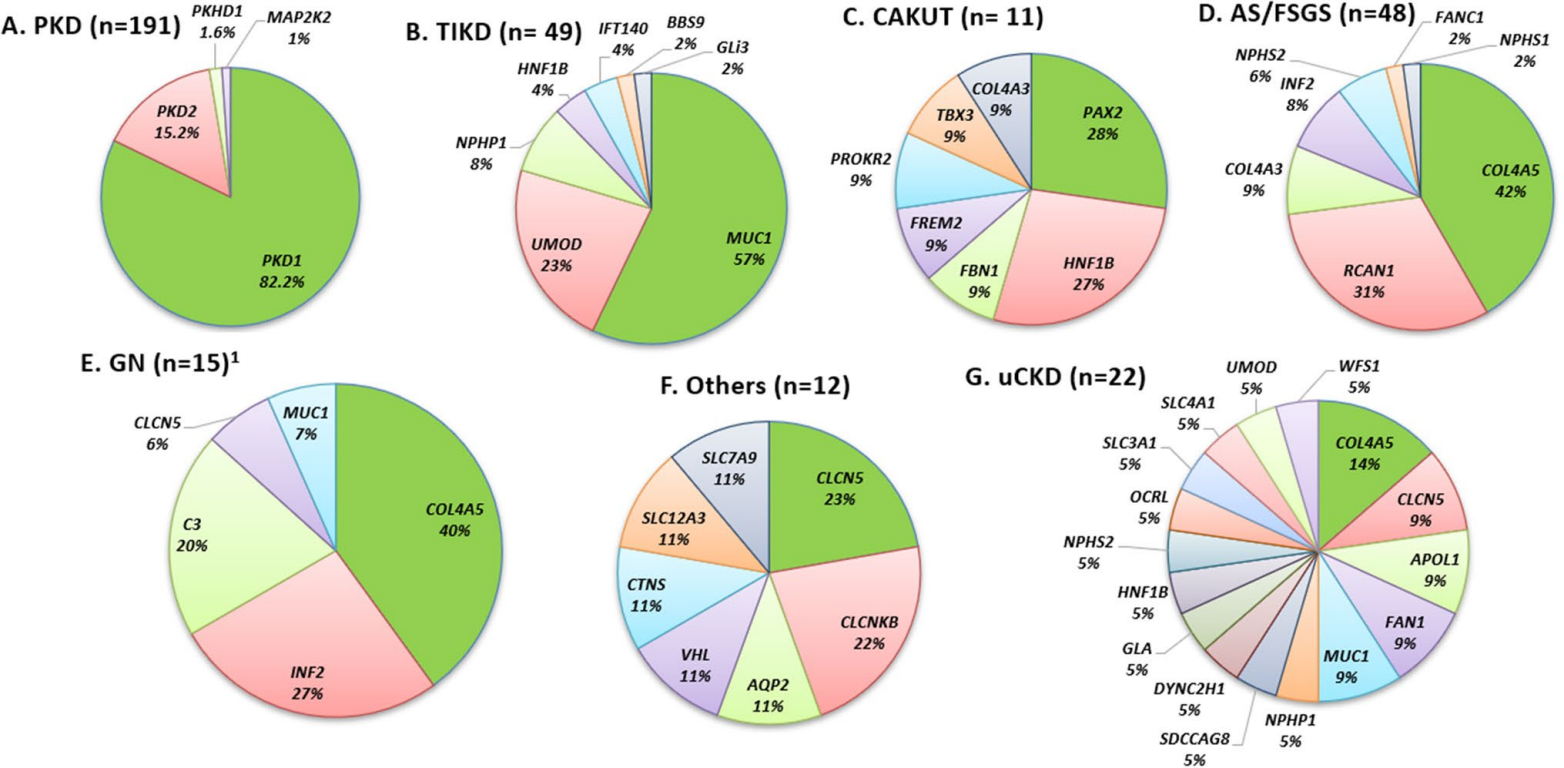
Disease-causing genes detected in the IKGP participants separated according to a priori diagnosis. *CAKUT* congenital anomalies of the kidney and urinary tract, *AS/FSGS* Alport syndrome/focal segmental glomerulosclerosis, *GN* glomerulonephritis, *PKD* polycystic kidney disease, *uCKD* chronic kidney disease of uncertain aetiology, *TIKD* tubulointerstitial kidney disease. ^1^In family F87, the proband had initial presentation of CKD stage 5, proteinuria, and family history of CKD, and referred with a priori diagnosis of GN. Proteinuria is thought to be a result of chronic changes rather than representing nature of the primary disease tubulointerstitial disease following the establishment of genetic diagnosis by *MUC1* sequencing and immunostaining for MUC1fs

According to the criteria adopted, we achieved a genetic diagnosis in 46.5% of the 501 families, corresponding to 51.4% of the 677 patients (Table 2, Supplementary Table S1). Two-thirds of patients with a reported family history of kidney disease achieved a genetic diagnosis (67.2% (336/500)) versus 31% (43/140) in patients with no family history of kidney disease (*P* =  < 0.001). Segregation analysis was required for 56 (9%) families. Amongst the 40 identified monogenic disorders, ACMG pathogenic or likely-pathogenic variants within four genes accounted for 70.7% of all identified causative variants; *PKD1* (*n* = 157/348; 123 families), *PKD2* (*n* = 29/348; 22 families), *MUC1* (*n* = 31/348; 10 families), and *COL4A5* (*n* = 29/348; 18 families). The remaining 29.3% of patients with a genetic diagnosis contained variants across a further 36 genes (Figs. 2 and 3).

**Table 2:** Distribution of diagnostic yield per a priori clinical diagnosis and sequencing technology

A priori Clinical diagnosis	Patients underwent gene Sequencing, *n*	Families per a priori clinical diagnosis, *n*	Clinical characteristics					Sequencing technology			Diagnostic yield	
			Median age at onset of ESKD, years (range)	CKD only in adulthood, *n* (%)	ESKD in adulthood, *n* (%)	ESKD in childhood, *n* (%)	Missing data of Renal Status	Gene-panel, *n* (%)	WES/WGS, *n* (%)	*MUC1* and *UMOD* sequencing, *n* (%)	Disease-causing variant per patients, *n* (%)	Disease-causing variant per families, *n* (%)
PKD	241	188	50 (6–83)	85 (35.3)	149 (61.8)	3 (1.2)	4 (1.7)	235 (97.5)	6 (2.5)	0 (0)	191 (79.2)	148 (78.7)
CAKUT	85	73	29 (3–66)	20 (23.5)	53 (62.4)	10 (11.8)	2 (2.3)	31 (36.5)	54 (63.5)	0 (0)	11 (12.8)	7 (9.6)
TIKD	75	39	34 (5–72)	23 (30.7)	33 (44)	7 (9.3)	12 (16)	12 (16)	23 (30.7)	40 (53.3)	49 (65.3)	18 (46.2)
Chronic GN	112	74	38.5 (9–68)	35 (31.3)	69 (61.6)	5 (4.5)	3 (2.6)	52 (46.4)	59 (52.7)	1 (0.9)	15 (13.4)	6 (8.1)
FSGS	39	20	40 (8–77)	15 (38.4)	18 (46.2)	5 (12.8)	1 (2.6)	13 (38.1)	26 (61.9)	0 (0)	24 (61.5)	7 (35)
AS	33	32	28 (15–77)	13 (39.4)	18 (54.5)	2 (6.1)	0 (0)	25 (63.4)	8 (36.6)	0 (0)	24 (72.7)	18 (56.2)
Tubular	4	4	38	3 (80)	1 (20)	0 (0)	0 (0)	2(50)	2(50)	0 (0)	4 (100)	4 (100)
uCKD	74	61	40 (9–71)	16 (21.6)	54(73)	4 (5.4)	0 (0)	34 (45.9)	38 (51.4)	2 (2.7)	22 (29.7)	19 (31.1)
Others	14	10	32 (6–62)	5 (35.8)	8 (57.1)	1 (7.1)	0 (0)	12 (85.7)	2 (14.3)	0 (0)	8 (57.1)	6 (50)
Total	677	501	30 (20–43)	215 (31.8)	403 (59.5)	37 (5.5)	22 (3.2)	416 (61.4)	218 (32.2)	43 (6.4)	348 (51.4)	233 (46.5)

*AS* Alport syndrome, *CAKUT* congenital anomalies of the kidney and urinary tract, *CKD* Chronic kidney disease, *ESKD* end stage kidney disease, *FSGS* focal segmental glomerulosclerosis, *MUC1* mucin 1 gene, *GN* glomerulonephritis, *PKD* polycystic kidney disease, *uCKD* CKD of uncertain aetiology, *TIKD* tubulointerstitial kidney disease, *WES* whole-exome sequencing, *WGS* whole-genome sequencing

In addition, variants of uncertain significance (VUS) considered to be clinically interesting by the MDT were detected in 11.8% (39/329; 32 families) of patients without a disease-causing variant. Segregation analysis is underway in 29 of these VUS families in an effort to reclassify these variants. In the remaining 236 families (47.1%), we were not able to obtain a genetic diagnosis.

### A priori diagnosis and identification of causative variants

*Polycystic kidney disease (PKD)*: PKD was the most prevalent a priori diagnosis (241/677, 35.6%). The a priori clinical diagnoses are listed in Table 1, with a detailed description in Supplementary Material. We identified a disease-causing variant in 78.7% (148/188) of families recruited with a priori diagnosis of PKD (Table 2), and targeted gene-panels were used as the primary sequencing technology. In 191 PKD patients with ACMG likely pathogenic/pathogenic variants, 157 carried a disease-causing variant in *PKD1*, and 29 in *PKD2* (Supplementary Table S1). Of the remaining five patients, three patients were found to have *PKHD1* variants associated with autosomal recessive PKD (ARPKD), and two carried *MAP2K2* variants associated with cardio-facio-cutaneous syndrome. Ten PKD families were identified to harbour clinically relevant VUS, whereas 30 families had no diagnostic results (16%).

*Non-Cystic GKD*: Among 313 families with non-cystic GKD, the diagnostic yield was 27.1%. The diagnostic yield varied within each diagnostic subgroup (Table 2, Supplementary Fig. S1).

Alport syndrome (AS)/focal segmental glomerulosclerosis (FSGS): Within the AS/FSGS cohort, we identified ACMG pathogenic/likely pathogenic variants within seven genes accounting for 48/72 individuals (66.7%). COL4A-related variants (*n* = 24) were the most frequent in this patient group and *COL4A5* predominated (*n* = 20, 41.6%). In one large family with autosomal dominant FSGS, we discovered a heterozygous NM_004414:p.Ile162Thr *RCAN1* variant, responsible for the patients’ FSGS [24]. Eight patients (3 families) were identified with disease-causing *INF2* variants, with a clear positive family history of proteinuric renal disease.

*Glomerulonephritis (GN) and IgA Nephropathy*: We identified a genetic diagnosis in 6 out of 74 families referred with chronic GN (8.1%). We detected three variants segregating in families with IgA nephropathy as reported by Stapleton et al. [22]. We did not identify a genetic diagnosis in any families with MPGN/C3GN. In family F87, the proband presented with advanced CKD stage 5, proteinuria and family history of CKD. Following re-examination of the kidney biopsy specimen, immunostaining for MUC1fs in urinary cell smears was positive confirming the diagnosis of *MUC1*-ADTKD, which was not suspected on clinical grounds before this study, hence correcting the clinical diagnosis from GN to ADTKD. Proteinuria was thought to be related to chronic changes.

*Tubulointerstitial kidney disease (TIKD)*: The well-established *MUC1* cytosine duplication variant was detected in 6 families (25 patients) with ADTKD. In four ADTKD families (7 patients) where a *MUC1* variant was not identified, we used non-invasive immunohistochemical urinary smear or kidney biopsies to confirm the presence of the frameshifted MUC1 protein (MUC1fs). Alternative genetic diagnoses were made in seven ADTKD families (18 patients); five with ADTKD-*UMOD* (12 patients), and two with ADTKD-*HNF1B* (6 patients). Amongst ADTKD families, TIKD predominated as an a priori diagnosis, but eight patients were initially referred with uCKD (*n* = 4), congenital anomalies of the kidney and urinary tract (CAKUT) (n = 3), or GN (*n* = 1). WES identified disease-causing variants in further eight patients with a priori diagnosis of TIKD and inconclusive biopsy findings.

*uCKD*: Amongst 61 families (74 patients) referred with a priori diagnosis of uCKD, 31.1% (19/61; 22 patients) were found to have a known monogenic disorder, bringing their diagnostic odyssey to an end, and emphasising the difficulties in making a clinical diagnosis in these very rare conditions without genetics support (Supplementary Table S1). In patients with a priori diagnosis of CAKUT, we detected 11 pathogenic/likely pathogenic variants in 11 individuals, corresponding to 7 of 73 families (9.6%). WES on three families with a child who had prune-belly syndrome did not reveal any underlying genetic cause.

*Kidney donors*: Three potential live kidney donors attended the clinic for screening due to a strong family history of CKD. In one case the potential donor was the sister of a patient with documented ADTKD-*MUC1,* while the other two patients had siblings with AS. In each case, we were able to confirm that the potential donors did not carry the disease-causing variants and were able to progress with living donation assessment.

### Diagnostic yield per platform and their clinical utility

WES resulted in a diagnostic rate of approximately 34%, whereas gene panel sequencing resulted in 56% (Fig. 2B). In ADTKD families, *MUC1* genotyping and targeted gene panel testing for *UMOD*, *REN* and *HNF1B* at the Broad resulted in a diagnostic rate of 70%. The further addition of urine smear analysis and tissue immunostaining for MUC1fs followed by sequencing of the entire *MUC1* gene increased this to 86% in ADTKD patients.

Excluding ADPKD cases, genetic testing confirmed the a priori diagnosis in 58% of individuals. Patients’ diagnoses were refined or a new diagnosis was made in 13% and 20% of non-ADPKD patients, respectively. In 15 of the 162 patients (9%), a new gene was identified as had been reported by Lane et al. [24] (Table 3). A genetic diagnosis facilitated a change in treatment plan in 28/162 (17.3%) patients, while a diagnostic kidney biopsy was deemed unnecessary in 13/162 (8%) patients as a direct result of a genetic diagnosis. In non-ADPKD patients, genetic results prompted reverse phenotyping such as targeted work-up for other associated extra-renal conditions in 51/162 (31.5%) and a further 70/162 (43.2%) patients had appropriate familial cascade testing.

**Table 3:** Summary of clinical outcomes of 162 patients (excluding cystic kidney disease) with a confirmed pathogenic diagnosis and the impact of genomic diagnosis on subsequent treatment

Diagnostic utility, *n* (%)	
Confirmed the a priori diagnosis	94 (58)
Refined the a priori diagnosis*	21 (13)
Established a new diagnosis	32 (19.8)
Novel candidate gene identified	15 (9.2)
Clinical utility, *n* (%)	
Negate Biopsy based on genomic diagnosis	13 (8)
Cascade tests/family counselling	70 (43.2)
Change pharmacological treatment	28 (17.3)
Additional assessment ordered to clarify extra-renal features—reverse phenotyping	51 (31.5)

*Genetic testing corrected/reclassified the a prior clinical diagnosis

Clinical lab validation of a genetic diagnosis was undertaken in 89/131 eligible patients, excluding those with ADPKD. Five did not return for clinical validation and 37 patients are awaiting return of results at the time of submission. At the beginning of the project, the median time from being evaluated in GKDC to return of validated results was 570.5 (IQR 317–1385) days, though the turnaround time decreased to 131 (IQR 100–205) days over the last 12 months of the study.

### Factors favouring diagnostic outcome

In multivariate logistic regression analysis, patients with causative variants were more than three-fold more likely to report family history of CKD (Odds ratio (OR) 3.69; 95% confidence interval (CI) = 2.1–6.48; *P* =  < 0.001). Patients with the underlying a priori diagnosis of PKD (OR: 14.9; 95% CI = 8.04–27.6; *P* =  < 0.001), TIKD (OR 4.7; 95% CI 2.15–10.3; *P* =  < 0.001), AS (OR 24.6; 95% CI 6.4–93.1; *P* =  < 0.001), FSGS (OR 6.3; 95% CI 2.3–17.4; *P* =  < 0.001), and uCKD (OR 7.6; 95% CI 1.7–32.4; *P* =  < 0.001) had a significantly higher frequency of diagnostic outcome relative to patients with GN. No statistical difference was observed regarding patients’ age (*P* = 0.246), age at disease onset < 18 years (*P* = 0.376), or sex (*P* = 0.471) between cases with or without diagnostic variants (Supplementary Tables S2 and S3).

## Discussion

Our study outlines the complexity of monogenic disorders, and the advantages of using genomic testing from diagnostic and clinical perspectives. We identified disease-causing variants in 46.5% (233/501) of GKD families (51.4% (348/677) of patients). The diagnostic yield in our cohort was consistent with several earlier studies [5, 14, 15], but higher than other studies [9, 10]. There are several factors that would explain our relatively high yield. First, we used a variety of genetic techniques to obtain a diagnosis. Earlier studies did not perform specialised genetic analysis for *MUC1* variants, which contributed to a significant number (8.9%) of diagnoses in our population. We also used specific clinical criteria to limit our population to a group of patients with a high risk of familial kidney disease. Similar to other studies [3, 9–11, 15], the presence of family history of CKD and the underlying a priori diagnosis were the two most significant predictors of a genetic diagnosis. In addition, a large proportion of the studied patients had clinically suspected PKD, which is known to have a high rate of genetic diagnosis. A targeted gene panel [23], designed to achieve high coverage of *PKD1* and *PKD2* was utilised in patients with an a priori diagnosis of PKD, which had considerable diagnostic utility (78.7%) in this population. However, studies have reported higher diagnostic rates at 86–94% [29–31], yet our relatively low diagnostic rate can be justified by the broad a priori definition which we adopted, in whom one-fifth of our patients reported no family history.

Employing WES, the diagnostic yield of around 34% in our adult cohort was comparable with a recent WES study by Jayasinghe et al. [9], which reported a genetic diagnosis in 80/204 (39%) patients across a spectrum of renal phenotype subcategories. In contrast to our study which exclusively involved adult patients, around 40% of the patients reported by Jayasinghe et al. were < 18 years of age.

Up to 10% of total solved cohort had a *COL4*-related genetic diagnosis, a prominent, yet often unsuspected cause of GKD in adults, which correlated with high diagnostic yield in the AS cohort (56.2%). A diagnostic yield of 35% was obtained using genomic sequencing in the FSGS cohort. Several large studies of adult patients with primary FSGS achieved diagnostic yields ranging from 29 to 37% and 12–30% for familial [32] and sporadic [32, 33] cases, respectively.

In Ireland, ADTKD accounts for 0.5% of ESKD patients [21]. The genetic diagnosis of ADTKD was made in a high percentage in our cohort. The ability to identify *MUC1* variants was important for the evaluation of GKD in our cohort, using both *MUC1* genetic sequencing and newer techniques to detect the mutant MUC1fs protein.

The ultimate goal of genetic testing is the potential for personalised medicine. We validated and returned most of the research-based testing [34]. In adults, similar to previous studies [9–12], our data demonstrate that GKDC results provide a precise genetic diagnoses with diagnostic and therapeutic implications (Table 3). Genetic testing has numerous other advantages including prognostics and ruling in or out familial kidney donors [35].

A primary weakness of this study was the observational nature of the methodology. A mono-ethnic cohort could limit the generalizability of the results. Also, we cannot exclude potential selection bias by having PKD as the main a priori diagnosis and using specific clinical criteria that limit our cohort to a highly specified group. Lastly, no licensed genetic counsellors or clinical geneticists were involved in reviewing our patients.

## Conclusions

In this large prospective cohort, the usage of various genomic testing strategies demonstrates their clinical application value, with a diagnostic yield over 50% supporting the advantageous clinical and therapeutic impact in adult patients with GKD. In our experience, an active renal genetic service requires a variety of genomic strategies and an integrated collaboration between clinical nephrologists and geneticists.

## Supplementary Information

Below is the link to the electronic supplementary material.

Supplementary file1 (DOCX 27 KB)

Supplementary file2 (DOCX 199 KB)
