# Supplementary material for: The utility of a genetic kidney disease clinic employing a broad range of genomic testing platforms: experience of the Irish Kidney Gene Project
Source: J Nephrol. 2022 Jan 31;35(6):1655–65. doi: 10.1007/s40620-021-01236-2 (PMC9300532; doi:10.1007/s40620-021-01236-2)
Supplement: Supplementary file 1 — Supplementary file1 (DOCX 27 KB) [file 40620_2021_1236_MOESM1_ESM.docx]

**Supplementary Material:**

In this prospective study, we summarise our experience of the Irish Kidney Gene Project (IKGP). We aimed to provide a national service to the network of nephrology units across the country to assist in the management of Genetic Kidney Disease (GKD). This study is approved by the Ethics Review Board of Beaumont Hospital and all patients have given explicit informed consent to participate.

The aims of our GKD clinic (GKDC) are:

1. To offer genetic testing to patients and their family members in whom a form of GKD is suspected.
2. To offer consultation and management advice in patients with rare potentially GKD.
3. To evaluate potential renal donors in those with a family history of kidney disease.
4. To assess affected or potentially affected family members of those in whom a genetic diagnosis had already been made.

All patients were invited to enrol in the IKGP and referred to attend the GKDC. Patients were reviewed and counselled in clinic by two trained nephrologists with interest in kidney diseases. A clinical diagnosis was made based on available clinical information and renal biopsy data. A family pedigree was drawn using a computational tool Progeny™ and pertinent clinical and biochemical information was extracted and stored in a research database. We pursued phenotype-driven genetic testing on the bases of the patient’s clinical presentation, thus in this study *a priori* clinical diagnoses have been grouped as per clinical and histological findings of the nephrologists’ referrals into one of the following categories:

- Cystic kidney disease involved those with autosomal dominant polycystic kidney disease (ADPKD) and patients with enlarged mutlicystic kidneys with or without family history.
- Tubulointerstitial kidney disease (TIKD) with biopsy findings of chronic tubulointerstital nephritis without a clear precipitating cause. Patients with a family history and kidney biopsy compatible with a diagnosis of autosomal dominant tubulointerstital kidney disease (ADTKD) were included.
- Chronic glomerulonephritis (GN) involved patients with membranoproliferative GN, including C3GN, haemolytic uraemic syndrome, familial IgA nephropathy, familial GN, and familial amyloid .
- Alport syndrome (AS) and focal segmental glomerulosclerosis (FSGS): Patients with clinical and / or histological suspicion of AS, in addition to those with nephrotic syndrome or nephrotic-range proteinuria and histopathological biopsy findings of FSGS with family history qualified for genetic testing.
- Congenital anomalies of the kidney and urinary tract (CAKUT) encompassing any abnormality within the kidneys or urinary tract involving number, size, shape, or anatomic position.
- Others involved potential kidney donors, renal tubulopathies, nephrolithiasis/ nephrocalcinosis, and other disorders like Alstrom disease, Von Hipple Lindau syndrome, and tuberous sclerosis.
- CKD of uncertain aetiology (uCKD) describes patients with inconclusive renal diagnosis following comprehensive workup with or without family history, including patients with bilateral small kidneys.

Because of the wide variety of patients presenting with different aetiologies and a variety of studies being performed under the umbrella of the IKGP, diagnostic genomic methods used to test individuals were diverse, therefore sequencing and bioinformatics analysis were carried out by D.M.C., S.C., C.S., K.K., A.J.B., M.Z., S.K., P.C.H., F.H., G.L.C., K.A.B. as reported elsewhere[1-8], and towards this, we present a summary of the IKGP generated data by the Next Generation Sequencing (NGS):

- Targeted NGS:
- We designed kidney disease panels, Roche SeqCap EZ Choice (with 227 known renal mendeliome genes) and Roche NimbleGen HeatSeq panel (11 gene panel) as published previously[1], to assist us in establishing a genetic diagnosis in a cohort of PKD patients (n= 235). Some of these patients (n=169) had been previously reported by Benson *et al**[**1]*.
- Gap-filling long-range (LR) PCR, Sanger sequencing and copy-number variants (CNVs): In ADPKD patients (n= 49) with no pathogenic variant detected on targeted NGS, LR-PCR and Sanger sequencing of *PKD1* and *PKD2* were performed. Identified CNVs using read-depth analysis were further confirmed using multiplex ligation-dependent probe amplification (n=5).
- Panels were also utilised in a number of patients’ phenotypes presenting with GKD in which we did not have a clear explanation as to the mechanism of disease (n= 130), as well as other patients with suspected familial CKD who presented to the IKDC (n=51). Gene list was previously published. Of these Genome Analysis Toolkit (GATK) Best Practices based pipelines were used to perform bioinformatic analyses.
- Whole exome-sequencing (WES): WES was targeted and sequenced as previously described [2, 9] and analysis of variants was restricted to evaluate more than 475 monogenic renal disease genes. The *a priori* clinical diagnoses were considered when evaluating for variants of match the phenotype. Variants were classified based on ACMG guidelines [10]. Only variants (SNVs/small Indels) in the coding region and the flanking intronic regions with minor allele frequency (MAF) <1% are evaluated. Known disease-causing variants were evaluated in up to ±30bp in flanking regions. MAFs are taken from public databases such as gnomAD. In this case, >99% of the targeted regions were covered by the minimum of 30 high-quality sequencing reads per base.
- We undertook WES on five cohorts of patients representing 193 patients from 140 families:
- We performed WES on 3 patients with a clinical diagnosis of prune belly syndrome with 2 families.
- We performed WES on 20 individuals from 10 families with familial IgA nephropathy.
- We performed WES on 20 individuals from 5 families with familial MPGN.
- We collaborated with Friedhelm Hildebrandt at Boston Children’s Hospital, Massachusetts, as previously described by Connaughton *et al**[**2]*, and undertook WES in 138 individuals from 114 families (RCSI).
- We performed WES, at CeGAT – Germany, on 12 individuals from 8 families that attended the IKDC between June and December 2020.
- Whole-genome sequencing (WGS): We carried out a linkage analysis through our collaboration with Rasheed Gbadegesin at Duke University School of Medicine, Durham, North Carolina and undertook WGS in 25 patients from 3 families to attempt to identify a pathogenic mutation. DNA libraries preparation, alignment, and sequencing using Illumina platforms had generated ≥120 Gb of raw data per sample as reported by Lane et al.[4]
- *MUC1* and MUC1fs testing: Gene testing for *MUC1* variants was performed at the Broad Institute, Massachusetts using techniques described elsewhere. UMOD mutational analysis was performed in all UMOD exons by the rare inherited kidney disease team of Wake Forest School of Medicine, Winston-Salem, North Carolina.
- All patients with an *a priori* diagnosis of ADTKD were referred to Anthony Bleyer at Wake Forest School of Medicine for *MUC1* and *UMOD* sequencing. *MUC1* genotyping for the C duplication within the variable number of tandem repeats (VNTR) and a gene panel for *UMOD*, *REN* and *HNF1B* were undertaken at the Broad Institute of Harvard and MIT, Cambridge, MA[8]. To date, we have tested 43 patients for *MUC1* or *UMOD*. Some of these results had been previously partially reported by Cormican *et al* [3]. When negative, immunostaining for MUC1fs in urinary cell smears or kidney biopsy[7] followed by entire MUC1 sequencing using either Illumina[7] or PacBio Single Molecule, Real-Time (SMRT) Sequencing[11] was performed at Charles University in Prague.

Where a genetic diagnosis was made, patients were invited to return to the clinic, counselled and had a further blood sample drawn for genomic sequencing of the identified variant, which was performed at an accredited clinical lab, Bioscientia - Germany. Only results replicated at the clinical lab were returned to patients. Negative results were communicated to patients and their physicians stating the fact that the current applied technologies could not define a Mendelian cause of their phenotype as part of a research test. Genomic results for ADPKD patients were not confirmed as part of an accredited test, as validation will not alter the clinical course. Nonetheless, two PKD patients requested accredited confirmation, as a precursor to pre-implantation genetic testing.

In cases of *MUC1*, no feasible accredited option was available for replication of clinical testing. Patients were informed about their results and counselled appropriately. These families had a family history and at least one kidney biopsy consistent with ADTKD.

**References:**

1. Benson, K.A., et al., *The genetic landscape of polycystic kidney disease in Ireland.* 2021. **29**(5): p. 827-838.

2. Connaughton, D.M., et al., *Monogenic causes of chronic kidney disease in adults.* 2019. **95**(4): p. 914-928.

3. Cormican, S., et al., *Autosomal dominant tubulointerstitial kidney disease (ADTKD) in Ireland.* 2019. **41**(1): p. 832-841.

4. Lane, B.M., et al., *A Rare Autosomal Dominant Variant in Regulator of Calcineurin Type 1 (RCAN1) Gene Confers Enhanced Calcineurin Activity and May Cause FSGS.* 2021.

5. Murray, S.L., et al., *Utility of Genomic Testing after Renal Biopsy.* 2020. **51**(1): p. 43-53.

6. Stapleton, C.P., et al., *An exome sequencing study of 10 families with IgA nephropathy.* 2020. **144**(2): p. 72-83.

7. Živná, M., et al., *Noninvasive Immunohistochemical Diagnosis and Novel MUC1 Mutations Causing Autosomal Dominant Tubulointerstitial Kidney Disease.* Journal of the American Society of Nephrology, 2018. **29**(9): p. 2418-2431.

8. Blumenstiel, B., et al., *Development and Validation of a Mass Spectrometry-Based Assay for the Molecular Diagnosis of Mucin-1 Kidney Disease.* Journal of Molecular Diagnostics, 2016. **18**(4): p. 566-71.

9. CeGAT, 2020.

10. Richards, S., et al., *Standards and guidelines for the interpretation of sequence variants: a joint consensus recommendation of the American College of Medical Genetics and Genomics and the Association for Molecular Pathology.* 2015. **17**(5): p. 405-423.

11. Vylet'al, P., et al., *Plasma Mucin-1 (CA15-3) Levels in Autosomal Dominant Tubulointerstitial Kidney Disease due to MUC1 Mutations.* American Journal of Nephrology, 2021: p. 1-10.
