# Supplementary material for: The utility of a genetic kidney disease clinic employing a broad range of genomic testing platforms: experience of the Irish Kidney Gene Project
Source: J Nephrol. 2022 Jan 31;35(6):1655–65. doi: 10.1007/s40620-021-01236-2 (PMC9300532; doi:10.1007/s40620-021-01236-2)
Supplement: Supplementary file 2 — Supplementary file2 (DOCX 199 KB) [file 40620_2021_1236_MOESM2_ESM.docx]

**Supplementary Table S1 ǀ** Identified disease-causing variants as per *a priori* diagnoses.

| **No.** | **Fam ID** | **ID** | **Sex** | ***A priori* clinical diagnosis** | **Age of First Diagnosis of CKD,**  **ESRD,**  **KTx**  **(years)** | **FHx** | **Gene**  **(Inheritance)** | **Zygosity** | **Variant** | **c.change**  **p.change** | **MAF^1^** | **ACMG** |
| --- | --- | --- | --- | --- | --- | --- | --- | --- | --- | --- | --- | --- |
| ***a priori* diagnosis - TIKD** | | | | | | | | | | | | |
|  | F80 | 8060 | F | NPHP | 21  21  24 | Yes | *NPHP1*  (AR; Biallelic) | Hom. | Frameshift | c.555_556insA  p.Pro186Thrfs*2 | 0 | Pathogenic  PVS1, PM2, PP1 |
|  | F80 | 8061 | M | NPHP | 11  12  12 | Yes | *NPHP1*  (AR; Biallelic) | Hom. | Frameshift | c.555_556insA  p.Pro186Thrfs*2 | 0 | Pathogenic  PVS1, PM2, PP1 |
|  | F389 | 10223 | M | NPHP | 8  16  16 | No | *NPHP1*  (AR; Biallelic) | Hom. | Non-synonymous SNV | c.1027G>A  p.Gly343Arg | 0.0001098 | Pathogenic  PVS1, PM2, PP3 |
|  | F324 | 10012 | M | Bilateral Echogenic Kidneys | 20  36  x | No | *BBS9*  (AR; Biallelic) | Hom. | Non-synonymous SNV | c.542C>G  p.Pro181Arg | 0.000003980 | Pathogenic  PP3, PM2, PP4, PM3 |
|  | F105 | 10558 | F | Bilateral small kidneys | 19  19  20 | Yes | *NPHP1*  (AR; Biallelic) | Hom. | Frameshift | c.555_556insA  p.Pro186Hisfs*2 | 0 | Pathogenic  PVS1, PM2, PP5 |
|  | F165 | 10080 | M | CKD – Gout, Renal Bx non-conclusive | 54  66  x | Yes | *UMOD*  (AD; Monoallelic) | Het. | Non-synonymous SNV | c.821A>G  p.Tyr274Cys | 0.000004084 | Likely Pathogenic  PM1, PP2, PM2, PP3 |
|  | F165 | 10081 | F | CKD Bland urine, Gout | 24  x  x | Yes | *UMOD*  (AD; Monoallelic) | Het. | Non-synonymous SNV | c.821A>G  p.Tyr274Cys | 0.000004084 | Likely Pathogenic  PM1, PP2, PM2, PP3 |
|  | F350 | 8164 | F | Normal sized cystic kidneys | 52  71  x | Yes | - | - | - | Urinary MUC1fs protein | - | Pathogenic  PVS1, PS3 |
|  | F350 | 10329 | M | CKD bland urine, Gout | 54  x  x  CKD G3b | Yes | - | - | - | Urinary MUC1fs protein | - | Pathogenic  PVS1, PS3 |
|  | F107 | 10425 | M | ADTKD | 43  46  47 | Yes | *MUC1*  (AD; Monoallelic) | Het. | Frameshift | c. ins(3n+1) in  VNTR  p. MUC1fs | 0 | Pathogenic  PVS1, PS1, PS3, PP1 |
|  | F107 | 10531 | F | ADTKD | 25  x  x  CKD G4 | Yes | *MUC1*  (AD; Monoallelic) | Het. | Frameshift | c. ins(3n+1) in  VNTR  p. MUC1fs | 0 | Pathogenic  PVS1, PS1, PS3, PP1 |
|  | F107 | 10554 | F | ADTKD | 35  35  36 | Yes | *MUC1*  (AD; Monoallelic) | Het. | Frameshift | c. ins(3n+1) in  VNTR  p. MUC1fs | 0 | Pathogenic  PVS1, PS1, PS3, PP1 |
|  | F13 | 10560 | F | Small, cystic kidneys | 5  12  12 | Yes | *IFT140*  (AR; Biallelic) | Hom. | Non-synonymous SNV | c.634G>A  p.Gly212Arg | 0.00005305 | Likely Pathogenic  PS1, PM2, PP3, PP4 |
|  | F13 | 10565 | M | Small, cystic kidneys | 2  27  28 | Yes | *IFT140*  (AR; Biallelic) | Hom. | Non-synonymous SNV | c.634G>A  p.Gly212Arg | 0.00005305 | Likely Pathogenic  PS1, PM2, PP3, PP4 |
|  | F859 | 11061 | M | ADTKD | 65  x  x | Yes | *-* | - | - | Urinary MUC1fs protein | - | Pathogenic  PVS1, PS3 |
|  | F860 | 11062 | F | ADTKD | 65  x  x  CKD G4 | Yes | *MUC1*  (AD; Monoallelic) | Het. | Frameshift | c. ins(3n+1) in  VNTR  p. MUC1fs | 0 | Pathogenic  PVS1, PS1, PS3, PP1 |
|  | F860 | 11063 | F | ADTKD | 35  x  x  CKD G4 | Yes | *MUC1*  (AD; Monoallelic) | Het. | Frameshift | c. ins(3n+1) in  VNTR  p. MUC1fs | 0 | Pathogenic  PVS1, PS1, PS3, PP1 |
|  | F860 | 11064 | M | ADTKD | 39  x  x  CKD G2 | Yes | *MUC1*  (AD; Monoallelic) | Het. | Frameshift | c. ins(3n+1) in  VNTR  p. MUC1fs | 0 | Pathogenic  PVS1, PS1, PS3, PP1 |
|  | F232 | 10060 | M | CKD – Renal Bx non-conclusive and Gout | 8  18  18 | Yes | *UMOD*  (AD; Monoallelic) | Het. | Non-synonymous SNV | c.1382C>A  p.Ala461Glu | 0 | Likely Pathogenic PM2, PP2, PP3, PP5, PP1 |
|  | F232 | 11218 | F | Non-proteinuric CKD, Renal Bx non-conclusive, Gout | 28  x  x  CKD G4 | Yes | *UMOD*  (AD; Monoallelic) | Het. | Non-synonymous SNV | c.1382C>A  p.Ala461Glu | 0 | Likely Pathogenic PM2, PP2, PP3, PP5, PP1 |
|  | FIN | 28101 | M | ADTKD | 51  53  54 | Yes | *UMOD*  (AD; Monoallelic) | Het. | Non-synonymous SNV | c.767G>A  p.Cys256Tyr | 0 | Likely Pathogenic  PM1, PP2, PM2, PP3, PP5 |
|  | FIN | 28107 | M | ADTKD | 18  33  33 | Yes | *UMOD*  (AD; Monoallelic) | Het. | Non-synonymous SNV | c.767G>A  p.Cys256Tyr | 0 | Likely Pathogenic  PM1, PP2, PM2, PP3, PP5 |
|  | FIN | 28108 | F | ADTKD | 22  x  x  CKD G4 | Yes | *UMOD*  (AD; Monoallelic) | Het. | Non-synonymous SNV | c.767G>A  p.Cys256Tyr | 0 | Likely Pathogenic  PM1, PP2, PM2, PP3, PP5 |
|  | FIN | 28109 | M | ADTKD | 35  39  40 | Yes | *UMOD*  (AD; Monoallelic) | Het. | Non-synonymous SNV | c.767G>A  p.Cys256Tyr | 0 | Likely Pathogenic  PM1, PP2, PM2, PP3, PP5 |
|  | F211 | 10552 | M | Renal Bx - TIN | 42  49  53 | Yes | *UMOD*  (AD; Monoallelic) | Het. | Non-synonymous SNV | c.317G>A  p.Cys106Tyr | 0 | Pathogenic  PM1, PM2, PM5, PP1, PP2, PP3, PP5 |
|  | F211 | 10553 | M | Renal Bx – TIN, Hyperuricemia | 42  x  x  CKD G5 | Yes | *UMOD*  (AD; Monoallelic) | Het. | Non-synonymous SNV | c.317G>A  p.Cys106Tyr | 0 | Pathogenic  PM1, PM2, PM5, PP1, PP2, PP3, PP5 |
|  | F211 | 28195 | M | Renal Bx - TIN | 49  x  x  CKD G2 | Yes | *UMOD*  (AD; Monoallelic) | Het. | Non-synonymous SNV | c.317G>A  p.Cys106Tyr | 0 | Pathogenic  PM1, PM2, PM5, PP1, PP2, PP3, PP5 |
|  | F386 | 10222 | F | ADTKD | 53  60  61 | Yes | *MUC1*  (AD; Monoallelic) | Het. | Frameshift | c. ins(3n+1) in  VNTR  p. MUC1fs | 0 | Pathogenic  PVS1, PS1, PS3, PP1 |
|  | F386 | 10248 | F | ADTKD | 33  43  43 | Yes | *MUC1*  (AD; Monoallelic) | Het. | Frameshift | c. ins(3n+1) in  VNTR  p. MUC1fs | 0 | Pathogenic  PVS1, PS1, PS3, PP1 |
|  | F386 | 10435 | F | ADTKD | 60  x  x  CKD G2 | Yes | *MUC1*  (AD; Monoallelic) | Het. | Frameshift | c. ins(3n+1) in  VNTR  p. MUC1fs | 0 | Pathogenic  PVS1, PS1, PS3, PP1 |
|  | F386 | 20-0705508 | M | ADTKD | N/A | Yes | *MUC1*  (AD; Monoallelic) | Het. | Frameshift | c. ins(3n+1) in  VNTR  p. MUC1fs | 0 | Pathogenic  PVS1, PS1, PS3, PP1 |
|  | F386 | 20-0705507 | F | ADTKD | N/A | Yes | *MUC1*  (AD; Monoallelic) | Het. | Frameshift | c. ins(3n+1) in  VNTR  p. MUC1fs | 0 | Pathogenic  PVS1, PS1, PS3, PP1 |
|  | F386 | 20-0705504 | F | ADTKD | N/A | Yes | *MUC1*  (AD; Monoallelic) | Het. | Frameshift | c. ins(3n+1) in  VNTR  p. MUC1fs | 0 | Pathogenic  PVS1, PS1, PS3, PP1 |
|  | F386 | 20-0705522 | F | ADTKD | 29  x  x  CKD G3 | Yes | *MUC1*  (AD; Monoallelic) | Het. | Frameshift | c. ins(3n+1) in  VNTR  p. MUC1fs | 0 | Pathogenic  PVS1, PS1, PS3, PP1 |
|  | F386 | 20-0705520 | M | ADTKD | 19  x  x  CKD G3a | Yes | *MUC1*  (AD; Monoallelic) | Het. | Frameshift | c. ins(3n+1) in  VNTR  p. MUC1fs | 0 | Pathogenic  PVS1, PS1, PS3, PP1 |
|  | F386 | 20-0705521 | M | ADTKD | 29  x  x  CKD G2 | Yes | *MUC1*  (AD; Monoallelic) | Het. | Frameshift | c. ins(3n+1) in  VNTR  p. MUC1fs | 0 | Pathogenic  PVS1, PS1, PS3, PP1 |
|  | F386 | 20-0705516 | F | ADTKD | N/A | Yes | *MUC1*  (AD; Monoallelic) | Het. | Frameshift | c. ins(3n+1) in  VNTR  p. MUC1fs | 0 | Pathogenic  PVS1, PS1, PS3, PP1 |
|  | F386 | 20-0705495 | M | ADTKD | 67  75  x | Yes | *MUC1*  (AD; Monoallelic) | Het. | Frameshift | c. ins(3n+1) in  VNTR  p. MUC1fs | 0 | Pathogenic  PVS1, PS1, PS3, PP1 |
|  | F386 | 20-0705506 | M | ADTKD | 41  46  52 | Yes | *MUC1*  (AD; Monoallelic) | Het. | Frameshift | c. ins(3n+1) in  VNTR  p. MUC1fs | 0 | Pathogenic  PVS1, PS1, PS3, PP1 |
|  | F386 | 20-0705512 | F | ADTKD | N/A  45  48 | Yes | *MUC1*  (AD; Monoallelic) | Het. | Frameshift | c. ins(3n+1) in  VNTR  p. MUC1fs | 0 | Pathogenic  PVS1, PS1, PS3, PP1 |
|  | F386 | 20-0705510 | F | ADTKD | N/A  43  44 | Yes | *MUC1*  (AD; Monoallelic) | Het. | Frameshift | c. ins(3n+1) in  VNTR  p. MUC1fs | 0 | Pathogenic  PVS1, PS1, PS3, PP1 |
|  | F386 | 20-0706279 | M | ADTKD | N/A  N/A  68 | Yes | *MUC1*  (AD; Monoallelic) | Het. | Frameshift | c. ins(3n+1) in  VNTR  p. MUC1fs | 0 | Pathogenic  PVS1, PS1, PS3, PP1 |
|  | D1 | N/A | F | ADTKD | N/A | Yes | *MUC1*  (AD; Monoallelic) | Het. | Frameshift | c. ins(3n+1) in  VNTR  p. MUC1fs | 0 | Pathogenic  PVS1, PS1, PS3, PP1 |
|  | D1 | N/A | F | ADTKD | N/A | Yes | *MUC1*  (AD; Monoallelic) | Het. | Frameshift | c. ins(3n+1) in  VNTR  p. MUC1fs | 0 | Pathogenic  PVS1, PS1, PS3, PP1 |
|  | D1 | N/A | M | ADTKD | N/A | Yes | *MUC1*  (AD; Monoallelic) | Het. | Frameshift | c. ins(3n+1) in  VNTR  p. MUC1fs | 0 | Pathogenic  PVS1, PS1, PS3, PP1 |
|  | F90 | 10563 | M | Familial Nephritis | 42  x  x  CKD G3a | Yes | *HNF1B*  (AD; Monoallelic) | Het. | Splicing | c.544+3_544+6  del 75% ESS | 0 | Likely Pathogenic  PSV1, PM2 |
|  | F90 | 10544 | F | Familial Nephritis | 37  40  x | Yes | *HNF1B*  (AD; Monoallelic) | Het. | Splicing | c.544+3_544+6  del 75% ESS | 0 | Likely Pathogenic  PSV1, PM2 |
|  | F402 | 8144 | F | NPHP | 0  12  13 | No | *GLi3*  (AD; Monoallelic) | Het. | Non-synonymous SNV | c.539G>A  p.Arg180Gln | 0.00005667 | Likely Pathogenic  PM1, PM2, PP2, PP3 |
|  | F106 | 28111 | M | ADTKD | N/A  30  31 | Yes | *MUC1*  (AD; Monoallelic) | Het. | Frameshift | c. ins(3n+1) in  VNTR  p. MUC1fs | 0 | Pathogenic  PVS1, PS1, PS3, PP1 |
| ***a priori* diagnosis - AS/FSGS** | | | | | | | | | | | | |
|  | F640 | 82 | M | FSGS / Low complement | 20  30  33 | Yes | *INF2*  (AD; Monoallelic) | Het. | Non-synonymous SNV | c.353T>A  p.Ile118Asn | 0 | Likely Pathogenic  PM1, PM2, PM5, PP3, PP5 |
|  |  |  |  |  |  |  | *C3*  (AD; Monoallelic) | Het. | Non-synonymous SNV | c.4534C>T  p.Arg1512Cys | 0.000007953 | Likely Pathogenic  PM2, PP1, PP2, PP3, PP4 |
|  | F640 | 83 | F | FSGS | 20  21  23 | Yes | *INF2*  (AD; Monoallelic) | Het. | Non-synonymous SNV | c.353T>A  p.Ile118Asn | 0 | Likely Pathogenic  PM1, PM2, PM5, PP3, PP5 |
|  | GF39 | 10426 | M | Alport syndrome | 20  47  51 | Yes | *COL4A5*  (XL) | Hemi | Splicing | c.2510-2A>G | 0.000005518 | Pathogenic PVS1, PP4, PP5 |
|  | GF39 | 10251 | M | Alport syndrome | 5  21  23 | Yes | *COL4A5*  (XL) | Hemi. | Splicing | c.2510-2A>G | 0.000005518 | Pathogenic PVS1, PP4, PP5 |
|  | F111 | 10052 | F | FSGS | 35  50  N/A | Yes | *RCAN1*  (AD; Monoallelic) | Het. | Non-synonymous SNV | c.485T>C  p.Ile162Thr | 0.0000007 | Pathogenic  PS3, PP3, PM1, PP1 |
|  | F111 | 10056 | M | FSGS | 29  27  28 | Yes | *RCAN1*  (AD; Monoallelic) | Het. | Non-synonymous SNV | c.485T>C  p.Ile162Thr | 0.0000007 | Pathogenic  PS3, PP3, PM1, PP1 |
|  | F111 | 10552 | M | Proteinuric CKD | N/A  N/A | Yes | *RCAN1*  (AD; Monoallelic) | Het. | Non-synonymous SNV | c.485T>C  p.Ile162Thr | 0.0000007 | Pathogenic  PS3, PP3, PM1, PP1 |
|  | F111 | 10486 | M | FSGS | 39  N/A  N/A | Yes | *RCAN1*  (AD; Monoallelic) | Het. | Non-synonymous SNV | c.485T>C  p.Ile162Thr | 0.0000007 | Pathogenic  PS3, PP3, PM1, PP1 |
|  | F111 | 10559 | M | FSGS | 48  N/A  71 | Yes | *RCAN1*  (AD; Monoallelic) | Het. | Non-synonymous SNV | c.485T>C  p.Ile162Thr | 0.0000007 | Pathogenic  PS3, PP3, PM1, PP1 |
|  | F111 | 10557 | M | FSGS | 66  77  x | Yes | *RCAN1*  (AD; Monoallelic) | Het. | Non-synonymous SNV | c.485T>C  p.Ile162Thr | 0.0000007 | Pathogenic  PS3, PP3, PM1, PP1 |
|  | F111 | 10229 | M | Nephrotic-range proteinuria | 45  x  x  CKD G1 | Yes | *RCAN1*  (AD; Monoallelic) | Het. | Non-synonymous SNV | c.485T>C  p.Ile162Thr | 0.0000007 | Pathogenic  PS3, PP3, PM1, PP1 |
|  | F111 | 10017 | F | Proteinuric CKD | 66  x  x  CKD G1 | Yes | *RCAN1*  (AD; Monoallelic) | Het. | Non-synonymous SNV | c.485T>C  p.Ile162Thr | 0.0000007 | Pathogenic  PS3, PP3, PM1, PP1 |
|  | F111 | 10126 | F | ASy | CKD G1  eGFR 95 mls | Yes | *RCAN1*  (AD; Monoallelic) | Het. | Non-synonymous SNV | c.485T>C  p.Ile162Thr | 0.0000007 | Pathogenic  PS3, PP3, PM1, PP1 |
|  | F111 | 10568 | F | ASy | N/A | Yes | *RCAN1*  (AD; Monoallelic) | Het. | Non-synonymous SNV | c.485T>C  p.Ile162Thr | 0.0000007 | Pathogenic  PS3, PP3, PM1, PP1 |
|  | F111 | 10231 | M | ASy | CKD G1 | Yes | *RCAN1*  (AD; Monoallelic) | Het. | Non-synonymous SNV | c.485T>C  p.Ile162Thr | 0.0000007 | Pathogenic  PS3, PP3, PM1, PP1 |
|  | F111 | 10125 | M | ASy | CKD G1 | Yes | *RCAN1*  (AD; Monoallelic) | Het. | Non-synonymous SNV | c.485T>C  p.Ile162Thr | 0.0000007 | Pathogenic  PS3, PP3, PM1, PP1 |
|  | F111 | 10233 | F | ASy | CKD G1 | Yes | *RCAN1*  (AD; Monoallelic) | Het. | Non-synonymous SNV | c.485T>C  p.Ile162Thr | 0.0000007 | Pathogenic  PS3, PP3, PM1, PP1 |
|  | F111 | 10234 | F | ASy | CKD G1 | Yes | *RCAN1*  (AD; Monoallelic) | Het. | Non-synonymous SNV | c.485T>C  p.Ile162Thr | 0.0000007 | Pathogenic  PS3, PP3, PM1, PP1 |
|  | F111 | 10586 | F | ASy | N/A | Yes | *RCAN1*  (AD; Monoallelic) | Het. | Non-synonymous SNV | c.485T>C  p.Ile162Thr | 0.0000007 | Pathogenic  PS3, PP3, PM1, PP1 |
|  | F100 | 10530 | F | FSGS | 30  40  45 | Yes | *COL4A5*  (XL; Monoallelic) | Het. | Non-synonymous SNV | c.2605G>A  p.Gly869Arg | 0 | Pathogenic  PM1, PP2, PM2, PP3, PM5, PP5 |
|  | F100 | 10616 | M | FSGS | 17  20  20 | Yes | *COL4A5*  (XL) | Hemi. | Non-synonymous SNV | c.2605G>A  p.Gly869Arg | 0 | Pathogenic  PM1, PP2, PM2, PP3, PM5, PP5 |
|  | KF4 | 22515 | F | Renal Bx non-conclusive, Gout | 52  55  56 | Yes | *INF2*  (AD; Monoallelic) | Het. | Non-synonymous SNV | c.653G>A  p.Arg218Gln | 0 | Pathogenic  PM2, PM5, PP3, PM1, PP5 |
|  | KF4 | 22516 | F | Renal Bx non-conclusive | 74  78 | Yes | *INF2*  (AD; Monoallelic) | Het. | Non-synonymous SNV | c.653G>A  p.Arg218Gln | 0 | Pathogenic  PM2, PM5, PP3, PM1, PP5 |
|  | F731 | 28172 | M | Alport syndrome | 22  x  x  CKD G1 | Yes | *COL4A5*  (XL) | Hemi. | Non-synonymous SNV | c.439G>A  p.Gly147Arg | 0 | Likely Pathogenic PM2, PP1, PP2, PP3, PP4 |
|  | F731 | 28173 | F | Haematuric CKD | 22  x  x  CKD G2 | Yes | *COL4A5*  (XL; Monoallelic) | Het. | Non-synonymous SNV | c.439G>A  p.Gly147Arg | 0 | Likely Pathogenic PM2, PP1, PP2, PP3, PP4 |
|  | F731 | 28174 | M | Alport syndrome | 23  x  x  CKD G3 | Yes | *COL4A5*  (XL) | Hemi. | Non-synonymous SNV | c.439G>A  p.Gly147Arg | 0 | Likely Pathogenic PM2, PP1, PP2, PP3, PP4 |
|  | F478 | 10376 | M | Alport syndrome | 18  25  26 | Yes | *COL4A5*  (XL) | Hemi. | Non-synonymous SNV | c.3310G>T  p.Gly1104Cys | 0 | Likely Pathogenic  PM2, PM3, PP3, PP4 |
|  | F982 | 11251 | M | FSGS | 10  x  x  CKD G3 | Yes | *NPHS2*  (AR; Biallelic) | Comp. Het. | Non-synonymous SNV | c.868G>A  p.Val290Met | 0.0001 | PathogenicPP5, PP3, PM2, PP2, PM1 |
|  |  |  |  |  |  |  |  |  | Non-synonymous SNV | c.413G>A  p.Arg138Gln | 0.0005969 | Pathogenic  PS4, PM2, PP3, PP2, PP5 |
|  | F81 | 10854 | F | FSGS | 8  13  13 | Yes | *NPHS2*  (AR; Biallelic) | Comp. Het. | Frameshift Deletion | c.855_856del  p.Gln285fs | 0.00006798 | Pathogenic  PVS1, PS4, PM2, PP5, PM3 |
|  |  |  |  |  |  |  |  |  | Non-synonymous SNV | c.413G>A  p.Arg138Gln | 0.0005969 | Pathogenic  PS4, PM2, PP3, PP2, PP5 |
|  | F81 | 10929 | M | FSGS | 8  14  15 | Yes | *NPHS2*  (AR; Biallelic) | Comp. Het. | Frameshift Deletion | c.855_856del  p.Gln285fs | 0.00006798 | Pathogenic  PVS1, PS4, PM2, PP5, PM3 |
|  |  |  |  |  |  |  |  |  | Non-synonymous SNV | c.413G>A  p.Arg138Gln | 0.0005969 | Pathogenic  PS4, PM2, PP3, PP2, PP5 |
|  | F976 | 10866 | F | Familial haematuria/ TBM | 28  x  x  CKD G1 | Yes | *COL4A5*  (XL; Monoallelic) | Het. | Non-synonymous SNV | c.3731G>A  p.Gly1244Asp | 0 | Pathogenic PM1, PP2, PP3, PP5 |
|  | F976 | 80730 | F | MPGN | 32  x  x  CKD G2 | Yes | *COL4A5*  (XL; Monoallelic) | Het. | Non-synonymous SNV | c.3731G>A  p.Gly1244Asp | 0 | Pathogenic PM1, PP2, PP3, PP5 |
|  | F612 | 8165 | M | Alport syndrome | 12  37  39 | Yes | *COL4A5*  (XL) | Hemi. | Non-synonymous SNV | c.1762G>A  p.Gly588Ser | 0 | Likely Pathogenic  PM2, PM3, PP2, PP3, PP4 |
|  | F58 | 10086 | M | Alport syndrome | 23  25  26 | Yes | *COL4A5*  (XL) | Hemi. | Splicing | c.1423+1G>T 100% ESS | 0 | Likely Pathogenic  PVS1, PM2 |
|  | KF7 | 22517 | F | Haematuria / Kidney Bx non-conclusive | 12  48  x | Yes | *COL4A3*  (AD; Monoallelic) | Het. | Non-synonymous SNV | c.2452G>A  p.Gly818Arg | 0 | Likely Pathogenic  PM2, PP3, PP2, PP5 |
|  | F275 | 10103 | M | Alport syndrome | 1  20  20 | Yes | *COL4A5*  (XL) | Hemi. | Splicing | c.4075-1G>A | 0 | Pathogenic PVS1, PM2, PP3, PP4 |
|  | F216 | 15862 | M | Alport syndrome | 11  20  23 | Yes | *COL4A3*  (AR; Biallelic) | Hom. | Stopgain | c.4825C>T  p.Arg1609Ter | 0.00001424 | Pathogenic  PVS1, PM2, PP5, |
|  | F403 | 10274 | M | Alport syndrome | 49  57  59 | Yes | *COL4A5*  (XL) | Hemi. | Non-frameshift Deletion | c.2959_2976del  p.987_992del | 0 | Likely Pathogenic PSV1, PM2, PM4, PP4,PP5 |
|  | F11 | 87085 | M | Haematuria / Kidney Bx IgM Nephropathy | 12  X  X  CKD G3a | Yes | *COL4A5*  (XL) | Hemi. | Non-synonymous SNV | c.3731G>A  p.Gly1244Asp | 0 | Pathogenic PM1, PM2, PP3, PP5 |
|  | F474 | 10375 | M | Bilateral small kidneys – Renal Bx FSGS | 2  14  15 | No | *FANCI*  (AR; Biallelic) | Hom. | Non-synonymous SNV | c.217A>T  p.Ile73Phe | 0 | Likely Pathogenic  PM2, PM6, PP3, PP4 |
|  | F681 | 10432 | M | Alport syndrome | 32  34  34 | Yes | *COL4A3*  (AD; Monoallelic) | Het. | Splicing | c.4253-1G>A | 0 | Likely Pathogenic  PVS1, PM2 |
|  | F693 | 10445 | M | Alport syndrome | 15  15  18 | No | *COL4A5*  (XL) | Hemi. | Frameshift Insertion | c.442dupC  p.G147fs | 0 | Pathogenic PVS1, PM2, PP4, PP5 |
|  | F705 | 10499 | M | Finnish Nephropathy | 0  0  1 | No | NPHS1  (AR; Biallelic) | Hom. | Splicing | c.2335-1G>A | 0 | Pathogenic PVS1, PM3, PP3 |
|  | CF7 | 22513 | M | Alport syndrome | 0  29  30 | Yes | *COL4A5*  (XL) | Hemi. | Non-synonymous SNV | c.2024G>T  p.Gly675Val | 0 | Likely Pathogenic  PM2, PM5, PP3, PP4 |
|  | F872 | 11080 | M | Alport syndrome / FSGS | 13  16  16 | Yes | *COL4A5*  (XL) | Hemi. | Non-synonymous SNV | c.3773G>A  p.Gly1258Asp | 0 | Likely Pathogenic PM1, PP2, PM2, PM5, PP3 |
|  | F822 | 11018 | F | Alport syndrome | 30  x  x  CKD G2 | Yes | *COL4A3*  (AD; Monoallelic) | Het. | Non-synonymous SNV | c.G3575A  p.Gly1192Glu | 0 | Likely Pathogenic  PM2, PP3, PP2, PP5 |
|  | F21 | 10220 | M | Alport syndrome | 17  20  24 | Yes | *COL4A5*  (XL) | Hemi. | Non-synonymous SNV | c.3509G>A  p.Gly170Asp | 0 | Likely pathogenic  PM1, PP2, PM2, PP5 |
|  | F21 | PF419 | F | Alport Syndrome | 37  43  46 | Yes | *COL4A5*  (XL; Monoallelic) | Het. | Non-synonymous SNV | c.3509G>A  p.Gly170Asp | 0 | Likely pathogenic  PM1, PP2, PM2, PP5 |
| ***a priori* diagnosis - CAKUT** | | | | | | | | | | | | |
|  | F307 | 10283 | F | VUR | 18  30  30 | Yes | *PAX2*  (AD; Monoallelic) | Het. | Non-synonymous SNV | c.70G>C  p.Gly24Arg | 0 | Likely Pathogenic  PM2, PM1, PP2, PM5, PP3 |
|  | F307 | 10285 | M | VUR | 42  46  48 | Yes | *PAX2*  (AD; Monoallelic) | Het. | Non-synonymous SNV | c.70G>C  p.Gly24Arg | 0 | Likely Pathogenic  PM2, PM1, PP2, PM5, PP3 |
|  | F307 | 10294 | F | Unilateral Renal Agenesis | 20  24  25 | Yes | *PAX2*  (AD; Monoallelic) | Het. | Non-synonymous SNV | c.70G>C  p.Gly24Arg | 0 | Likely Pathogenic  PM2, PM1, PP2, PM5, PP3 |
|  | F306 | 10092 | F | Reflux nephropathy – Familial CKD | 3  13  13 | Yes | *HNF1B*  (AD; Monoallelic) | Het. | Frameshift | c.1333_1334del  p.Ala445fs | 0 | Pathogenic  PM2, PP1, PP4 |
|  | F306 | 10403 | M | Reflux with renal cysts and Diabetes | 38  39  40 | Yes | *HNF1B*  (AD; Monoallelic) | Het. | Frameshift | c.1333_1334del  p.Ala445fs | 0 | Pathogenic  PM2, PP1, PP4 |
|  | F306 | 10404 | M | Reflux nephropathy – Familial CKD | 29  55  64 | Yes | *HNF1B*  (AD; Monoallelic) | Het. | Frameshift | c.1333_1334del  p.Ala445fs | 0 | Pathogenic  PM2, PP1, PP4 |
|  | N/A | 10096 | F | VUR | 0  x  x | No | *FBN1*  (AD; Monoallelic) | Het. | Stopgain | c.4888C>T  p.Gln1630Ter | 0 | Pathogenic  PVS1/ PM2/PP5 |
|  | F162 | 10099 | M | VUR | 50  51  x | Yes | *FREM2*  (AR; Biallelic) | Comp. Het. | Non-synonymous SNV | c.3661C>T  p.Pro1221Ser | 0 | Likely Pathogenic PP1, PM3, PM2, PP3 |
|  |  |  |  |  |  |  |  |  |  | c.2533C>T  p.His845Tyr | 0 | Likely Pathogenic PP1, PM3, PM2, PP3 |
|  | F397 | 10256 | M | Haematuria with bilateral hypodysplasia | 3  29  30 | No | *COL4A3*  (AD; Monoallelic) | Het. | Non-synonymous SNV | c.4981C>T  p.Arg1661Cys | O | Likely Pathogenic  PP2, PP5, PM2 |
|  | F410 | 10312 | F | CAKUT | 0  14  15 | No | *PROKR2*  (AD; Monoallelic) | Het. | Non-synonymous SNV | c.332T>G  p.Met111Arg | 0 | Likely Pathogenic PP1, PM2, PP3, PP2, PP4 |
|  | F606 | 10383 | M | CAKUT | 0  12  12 | No | *TBX3*  (AD; Monoallelic) | Het. | Frameshift | c.915del  p.Asp305Glufs*18 | 0 | Pathogenic  PVS1, PM2 |
| ***a priori* diagnosis – uCKD** | | | | | | | | | | | | |
|  | F774 | 10974 | F | uCKD | N/A | No | *COL4A3*  (AR; Biallelic) | Comp. Het. | Frameshift Deletion | c.661_662del  p.Arg221fs | 0 | Likely Pathogenic  PVS1, PM2, PP5 |
|  |  |  |  |  |  |  |  |  | Stopgain | c.1591C>T  p.Gln531Ter | 0 |  |
|  | F982 | 11247 | F | uCKD | 16  44  x | Yes | *NPHS2*  (AR; Biallelic) | Comp. Het. | Non-synonymous SNV | c.868G>A  p.Val290Met | 0.0001 | Pathogenic  PP5, PP3, PM2, PP2, PM1 |
|  |  |  |  |  |  |  |  |  | Non-synonymous SNV | c.413G>A  p.Arg138Gln | 0.0005969 | Pathogenic  PS4, PM2, PP3, PP2, PP5 |
|  | F306 | 28192 | F | uCKD | 52  69  72 | Yes | *HNF1B*  (AD; Monoallelic) | Het. | Frameshift | c.1333_1334del  p.Ala445fs | 0 | Pathogenic  PM2, PP1, PP4 |
|  | F88 | 10538 | F | Bilateral small kidneys | 45  47  48 | Yes | *FAN1*  (AR; Biallelic) | Comp. Het. | Stopgain | c.2590C>T  p.Gln864Ter | 0 | Likely Pathogenic  PVS1, PM2 |
|  |  |  |  |  |  |  |  |  | Frameshift | c.2774_2775delTT  p.Leu925fs | 0 | Pathogenic  PVS1, PM2, PP5 |
|  | F88 | 10547 | M | Bilateral small kidneys | 44  45  45 | Yes | *FAN1*  (AR; Biallelic) | Comp Het. | Stopgain | c.2590C>T  p.Gln864Ter | 0 | Likely Pathogenic  PVS1, PM2 |
|  |  |  |  |  |  |  |  |  | Frameshift | c.2774_2775delTT  p.Leu925fs | 0 | Pathogenic  PVS1, PM2, PP5 |
|  | F141 | 11260 | M | uCKD | 49  49  x | Yes | *COL4A5*  (XL) | Hemi. | Non-synonymous SNV | c.3427G>A  p.Gly1143Ser | 0 | Pathogenic  PM1, PP2, PM2, PM5, PP3, PP5 |
|  | F309 | 10590 | M | uCKD | N/A  67  x | Yes | - | - | - | Kidney Biopsy MUC1fs protein | - | Pathogenic  PVS1, PS3 |
|  | F322 | 10011 | F | Bilateral small kidneys, Retinitis Pigmentosa | 62  70  x | No | *DYNC2H1* (AR; Biallelic) | Comp. Het. | Non-synonymous SNV | c.12431C>G  p.Pro4144Arg | 0 | VUS  PM2, PP5 |
|  |  |  |  |  |  |  |  |  | Splicing | c.10063+2T>G  100% ESS | 0 | Pathologic PVS1, PM2, PP5 |
|  | N/A | 10078 | F | Renal Bx Indeterminate | 25  x  x | No | *SLC3A1*  (AD; Monoallelic) | Het. | Non-synonymous SNV | c.1799G>A  p.Gly600Glu | 0.00007437 | Likely Pathogenic  PM1, PM2, PP2, PP3, PP4 |
|  | F193 | 10083 | F | uCKD | 20  x  x | No | *UMOD*  (AD; Monoallelic) | Het. | Non-synonymous SNV | c.280T>C  p.Cys94Arg | 0 | Likely Pathogenic  PM1, PP2, PM2, PP3 |
|  | F198 | 10102 | F | uCKD; Hypertension | 36  x  x | Yes | *WFS1*  (AD; Monoallelic) | Het. | Non-synonymous SNV | c.2654C>T  p.Pro885Leu | 0 | Likely Pathogenic  PM3, PP2, PP5 |
|  | F318 | 10150 | M | Bilateral small kidneys, Intellectual disability, seizures and finger and wrist swelling | 41  x  x | Yes | *OCRL*  (XL) | Hemi. | Non-synonymous SNV | c.1567G>A  p.Asp523Asn | 0 | Pathogenic  PM1, PP2, PM2, PM5, PP3, PP5 |
|  | F313 | 10515 | F | Bilateral small kidneys | 60  x  x | Yes | *CLCN5*  (XL; Monoallelic) | Het. | Non-synonymous SNV | c.925C>T  p.Arg309Cys | 0.00003525 | Likely Pathogenic  PM2, PP2, PP3 |
|  | F182 | 10602 | M | Bilateral small kidneys | 51  51  55 | Yes | *CLCN5*  (XL) | Hemi. | Frameshift | c.1938del  p.Phe646LeufsTer10 | 0 | Likely Pathogenic  PVS1, PM2 |
|  | F804 | 10992 | M | uCKD; Hypertensive kidney disease | 41  41  50 | Yes | *APOL1* | Hom. | G1/G1 | c.1072A>G  p.Ser358Gly | 0.00001193 | Likely Pathogenic  PS4, PP5 |
|  |  |  |  |  |  |  |  |  |  | c.1200T>G  p.Ile400Met | 0.01554 |  |
|  | F101 | 28110 | F | uCKD; Pyelonephritis | N/A  28  29 | Yes | *-* | - | - | Urinary MUC1fs protein | - | Pathogenic  PVS1, PS3 |
|  | F725 | 28152 | M | uCKD; presumed Fabry’s Disease | 23  38  38 | Yes | *GLA*  (XL) | Hemi. | Stopgain | c.132G>A  p.Trp44Ter | 0 | Pathogenic  PVS1, PM2, PP5 |
|  | F1007 | WES02 | F | uCKD; inconclusive kidney Biopsy | 19  22  24 | Yes | *SDCCAG8*  (AR; Biallelic) | Comp. Het. | Stopgain | c.484C>T  p.Gln162Ter | 0.000003980 | Pathogenic  PVS1, PM2 |
|  |  |  |  |  |  |  |  |  | Stopgain | c.696T>G  p.Tyr232Ter | 0.000003986 | Pathogenic  PVS1, PM2, PP5 |
|  | F1014 | WES07 | M | uCKD; Increased BMI, left eye diminution of vision, abnormal gait; Extensive Interstitial fibrosis | 16  x  x | No | *NPHP1*  (AR; Biallelic) | Hom. | Splicing | c.1438-1G>A | 0.000004064 | Likely Pathogenic  PVS1, PM3 |
|  | F673 | 10453 | M | Hypertensive CKD | 15  26  28 | Yes | *COL4A5*  (XL) | Hemi. | Non-synonymous SNV | c.439G>A  p.Gly147Arg | 0 | Likely Pathogenic PM2, PP1, PP2, PP3, PP4 |
|  | F1018 | WES17 | M | uCKD | 21  x  x  CKD G2 | Yes | *COL4A5*  (XL) | Hemi. | Non-synonymous SNV | c.3731G>A  p.Gly1244Asp | 0 | Pathogenic PM1, PM2, PP3, PP5 |
|  | F946 | 10839 | F | uCKD; Hx of nephrolithiasis | 40  58  60 | Yes | *SLC4A1*  (AD; Monoallelic) | Het. | Non-synonymous SNV | c.1765C>T  p.Arg589Cys | 0 | Likely Pathogenic  PM2, PM5, PP3, PP5 |
| ***a priori* diagnosis – Chronic GN** | | | | | | | | | | | | |
|  | F640 | 2008 | M | Microscopic  Haematuria, Low complement (C3) levels | 20  x  x | Yes | *C3*  (AD; Monoallelic) | Het. | Non-synonymous SNV | c.4534C>T  p.Arg1512Cys | 0.000007953 | Likely Pathogenic  PM2, PP1, PP2, PP3, PP4 |
|  | F640 | 28124 | F | Microscopic  Haematuria, Low complement (C3) levels | 56  x  x | Yes | *C3*  (AD; Monoallelic) | Het. | Non-synonymous SNV | c.4534C>T  p.Arg1512Cys | 0.000007953 | Likely Pathogenic  PM2, PP1, PP2, PP3, PP4 |
|  | F640 | 28119 | M | Microscopic  Haematuria, Low complement (C3) levels | 55  x  x | Yes | *C3*  (AD; Monoallelic) | Het. | Non-synonymous SNV | c.4534C>T  p.Arg1512Cys | 0.000007953 | Likely Pathogenic  PM2, PP1, PP2, PP3, PP4 |
|  | F677 | 10456 | F | Familial Fibrillary GN | 23  N/A  N/A | Yes | *INF2*  (AD; Monoallelic) | Het. | Non-synonymous SNV | c.640C>T  p.Arg214Cys | 0.000004031 | Pathogenic  PM1, PM2, PM5, PP3, PP4, PP5 |
|  | F677 | 10482 | M | Familial Fibrillary GN | 26  37  37 | Yes | *INF2*  (AD; Monoallelic) | Het. | Non-synonymous SNV | c.640C>T  p.Arg214Cys | 0.000004031 | Pathogenic  PM1, PM2, PM5, PP3, PP4, PP5 |
|  | F677 | 10483 | M | Familial Fibrillary GN | 24  44  x | Yes | *INF2*  (AD; Monoallelic) | Het. | Non-synonymous SNV | c.640C>T  p.Arg214Cys | 0.000004031 | Pathogenic  PM1, PM2, PM5, PP3, PP4, PP5 |
|  | F677 | 10484 | M | Familial Fibrillary GN | 28  x  x  CKD G2 | Yes | *INF2*  (AD; Monoallelic) | Het. | Non-synonymous SNV | c.640C>T  p.Arg214Cys | 0.000004031 | Pathogenic  PM1, PM2, PM5, PP3, PP4, PP5 |
|  | F141 | 10801 | F | Familial IgA Nephropathy | 19  x  x  CKD G1 | Yes | *COL4A5*  (XL; Monoallelic) | Het. | Non-synonymous SNV | c.3427G>A  p.Gly1143Ser | 0 | Pathogenic  PM1, PP2, PM2, PM5, PP3, PP5 |
|  | F141 | 10121 | M | Familial IgA Nephropathy | 46  x  x  CKD G1 | Yes | *COL4A5*  (XL) | Hemi. | Non-synonymous SNV | c.3427G>A  p.Gly1143Ser | 0 | Pathogenic  PM1, PP2, PM2, PM5, PP3, PP5 |
|  | F141 | 10087 | M | Familial IgA Nephropathy | 12  26  39 | Yes | *COL4A5*  (XL) | Hemi. | Non-synonymous SNV | c.3427G>A  p.Gly1143Ser | 0 | Pathogenic  PM1, PP2, PM2, PM5, PP3, PP5 |
|  | F141 | 10169 | M | Familial IgA Nephropathy | 40  x  x  CKD G3a | Yes | *COL4A5*  (XL) | Hemi. | Non-synonymous SNV | c.3427G>A  p.Gly1143Ser | 0 | Pathogenic  PM1, PP2, PM2, PM5, PP3, PP5 |
|  | F141 | 10014 | M | Familial IgA Nephropathy | 42  x  x  CKD G1 | Yes | *COL4A5*  (XL) | Hemi. | Non-synonymous SNV | c.3427G>A  p.Gly1143Ser | 0 | Pathogenic  PM1, PP2, PM2, PM5, PP3, PP5 |
|  | F87 | 10536 | M | CKD - Non conclusive biopsy - TMA | 18  18  21 | Yes | *-* | - | - | Urinary MUC1fs protein | - | Pathogenic  PVS1, PS3 |
|  | F708 | 28116 | M | IgA Nephropathy with sensorineural deafness | 17  x  x  CKD G4 | Yes | *CLCN5*  (XL) | Hemi. | Stopgain | c.275G>A  p.Trp92Ter | 0 | Likely Pathogenic PVS1, PM2, BP4 |
|  | F424 | 10210 | F | Haematuria and proteinuria – Kidney Bx: MPGN | 51  Pre-emptive Tx  54 | N/A | *COL4A5*  (XL; Monoallelic) | Het. | Non-synonymous SNV | c.3310G>T  p.Gly1104Cys | 0 | Likely Pathogenic  PM2, PM3, PP3, PP4 |
| ***a priori* diagnosis – Others**^2^ | | | | | | | | | | | | |
|  | F656 | 8180 | M | Bartter Syndrome - Hypokalemic  metabolic  alkalosis | 3  x  x  CKD G1 | No | *CLCNKB*  (AR; Biallelic) | Hom. | Stopgain | c.226C>T  p.Arg76Ter | 0.00001195 | Pathogenic  PVS1, PM2, PP5 |
|  | F323 | 10132 | F | Hypokalemic Metabolic alkalosis | 17  38  38 | Yes | *CLCNKB*  (AR; Biallelic) | Hom. | Stopgain | c.226C>T  p.Arg76Ter | 0.00001195 | Pathogenic  PVS1, PM2, PP5 |
|  | F610 | 28128 | M | Ricketts and nephrocalcinosis  Dent's disease | 16  22  x | Yes | *CLCN5*  (XL) | Hemi. | Non-synonymous SNV | c.941C>T  p.Ser314Leu | 0 | Likely pathogenic PM2, PP3, PP5 |
|  | F935 | 11174 | M | Nephrocalcinosis and family history of renal failure | 55  59  60 | Yes | *CLCN5*  (XL) | Hemi. | Splicing | c.166-2A>T | 0 | Likely Pathogenic PVS1, PM2 |
|  | F945 | 10838 | M | Malignant hypertension, blindness | 17  31  34 | Yes | *VHL*  (AD; Monoallelic) | Het. | Non-synonymous SNV | c.562C>G  p.Leu188Val | 0.00001989 | Pathogenic  PM1, PP2, PM5, PP3, PP5 |
|  | F1025 | WES14 | F | Hypokalemic metabolic alkalosis with low Mg+ – worsened during pregnancy | 25  x  x  CKD G1 | Yes | *SLC12A3*  (AR; Biallelic?) | Comp. Het. | Non-synonymous SNV | c.2221G>A  p.Gly741Arg | 0.0003701 | Pathogenic  PM2, PM1, PP2, PP3, PP5 |
|  |  |  |  |  |  |  |  |  | - | Other variant was not detected by NGS | - | - |
|  | F903 | 11126 | F | Recurrent cysteine nephrolithiasis | 14  x  x  CKD G1 | Yes | *SLC7A9*  (AR; Biallelic) | Comp. Het. | Non-synonymous SNV | c.368C>T  p.Thr123Met | 0.002 | Likely Pathogenic  PS4, PM1, PP4 |
|  |  |  |  |  |  |  |  |  | Non-synonymous SNV | c.920T>G  p.Leu307Arg | 0.0007 | VUS  PM2, PP3 |
|  | F886 | 10846 | F | Proteinuria, glucosuria, and Photophobia due to Cystinosis | Birth  x  x  CKD G3a | Yes | *CTNS*  (AR; Biallelic) | Hom. | Stopgain | c.414G>A  p.Trp138Ter | 0.00004772 | Pathogenic  PVS1, PM2, PP5 |
|  | F413 | 10278 | M | Dysplastic Kidneys | 0  10  12 | No | *AQP2*  (AD; Monoallelic) | Het. | Non-synonymous SNV | c.782C>T  p.Ser261Leu | 0.00001711 | Likely Pathogenic PM2, PM1, PP2, PP3 |
| ***a priori* diagnosis - Cystic Kidney Diseases** | | | | | | | | | | | | |
|  | TF65 | 10205 |  | ADPKD | 16  39  41 | Yes | *PKD1*  (AD; Monoallelic) | Het. | Splicing | c.12445-1G>A | 0 | Likely Pathogenic  PVS1, PM2 |
|  | TF65 | 11227 |  | ADPKD | 4  x  x  CKD G1 | Yes | *PKD1*  (AD; Monoallelic) | Het. | Splicing | c.12445-1G>A | 0 | Likely Pathogenic  PVS1, PM2 |
|  | TF65 | 11228 |  | ADPKD | 15  x  x  CKD G1 | Yes | *PKD1*  (AD; Monoallelic) | Het. | Splicing | c.12445-1G>A | 0 | Likely Pathogenic  PVS1, PM2 |
|  | F567 | 10855 | F | ADPKD | 30  x  x  CKD G5 | Yes | *PKD1*  (AD; Monoallelic) | Het. | Stopgain | c.4306C>T  p.Arg1436Ter | 0 | Pathogenic  PVS1, PM2, PP4, PP5 |
|  | F567 | 10856 | F | ADPKD | 30  x  x  CKD G1 | Yes | *PKD1*  (AD; Monoallelic) | Het. | Stopgain | c.4306C>T  p.Arg1436Ter | 0 | Pathogenic  PVS1, PM2, PP4, PP5 |
|  | F567 | 10857 | F | ADPKD | 30  47  x | Yes | *PKD1*  (AD; Monoallelic) | Het. | Stopgain | c.4306C>T  p.Arg1436Ter | 0 | Pathogenic  PVS1, PM2, PP4, PP5 |
|  | F692 | 10863 | M | ADPKD | 40  59  60 | Yes | *PKD2*  (AD; Monoallelic) | Het. | Stopgain | c.602G>A  p.Trp201Ter | 0 | Pathogenic  PVS1, PM2, PP3, PP4, PP5 |
|  | F692 | 10446 | F | ADPKD | 28  x  x | Yes | *PKD2*  (AD; Monoallelic) | Het. | Stopgain | c.602G>A  p.Trp201Ter | 0 | Pathogenic  PVS1, PM2, PP3, PP4, PP5 |
|  | TF90 | 11232 | F | ADPKD | 7  x  x  CKD G1 | Yes | *PKD1*  (AD; Monoallelic) | Het. | Frameshift Insertion | c.912_913insG  p.Trp305Valfs*66 | 0 | Pathogenic  PVS1, PM2 |
|  | TF90 | 11233 | F | ADPKD | 36  x  x  CKD G5 | Yes | *PKD1*  (AD; Monoallelic) | Het. | Frameshift Insertion | c.912_913insG  p.Trp305Valfs*66 | 0 | Pathogenic  PVS1, PM2 |
|  | TF90 | 11236 | F | ADPKD | 11  x  x  CKD G1 | Yes | *PKD1*  (AD; Monoallelic) | Het. | Frameshift Insertion | c.912_913insG  p.Trp305Valfs*66 | 0 | Pathogenic  PVS1, PM2 |
|  | TF90 | 8126 | F | ADPKD | 35  48  50 | Yes | *PKD1*  (AD; Monoallelic) | Het. | Frameshift Insertion | c.912_913insG  p.Trp305Valfs*66 | 0 | Pathogenic  PVS1, PM2 |
|  | F355 | 10268 | M | ADPKD | 15  47  49 | Yes | *PKD1*  (AD; Monoallelic) | Het. | Splicing | c.2097+5G>A | 0 | Pathogenic  PVS1, PP1, PP4 |
|  | F355 | 8196 | F | ADPKD | 12  x  x | Yes | *PKD1*  (AD; Monoallelic) | Het. | Splicing | c.2097+5G>A | 0 | Pathogenic  PVS1, PP1, PP4 |
|  | F605 | 11014 | M | ADPKD | 19  45  45 | Yes | *PKD1*  (AD; Monoallelic) | Het. | Stopgain | c.9298C>T  p.Gln3100Ter | 0 | Pathogenic  PVS1, PM2, PP4 |
|  | F605 | 8157 | M | ADPKD | 34  51  51 | Yes | *PKD1*  (AD; Monoallelic) | Het. | Stopgain | c.9298C>T  p.Gln3100Ter | 0 | Pathogenic  PVS1, PM2, PP4 |
|  | F649 | 10876 | F | ADPKD | 40  50  53 | Yes | *PKD1*  (AD; Monoallelic) | Het. | Stopgain | c.11343C>G  p.Tyr3781Ter | 0 | Pathogenic  PVS1, PM2, PP1, PP4, PP5 |
|  | F649 | 10630 | M | ADPKD | 32  51  x | Yes | *PKD1*  (AD; Monoallelic) | Het. | Stopgain | c.11343C>G  p.Tyr3781Ter | 0 | Pathogenic  PVS1, PM2, PP1, PP4, PP5 |
|  | F649 | 10654 | F | ADPKD | 23  x  x  CKD G2 | Yes | *PKD1*  (AD; Monoallelic) | Het. | Stopgain | c.11343C>G  p.Tyr3781Ter | 0 | Pathogenic  PVS1, PM2, PP1, PP4, PP5 |
|  | F649 | 10669 | M | ADPKD | 10  50  52 | Yes | *PKD1*  (AD; Monoallelic) | Het. | Stopgain | c.11343C>G  p.Tyr3781Ter | 0 | Pathogenic  PVS1, PM2, PP1, PP4, PP5 |
|  | F618 | 10629 | M | ADPKD | 29  33  35 | Yes | *PKD1*  (AD; Monoallelic) | Het. | Inframe deletion | c.858_932del  p.Gly287_Ser311del | 0 | Likely pathogenic (PM2, PM4, PP1, PP4) |
|  | F618 | 10631 | F | ADPKD | 53  53  x | Yes | *PKD1*  (AD; Monoallelic) | Het. | Inframe deletion | c.858_932del  p.Gly287_Ser311del | 0 | Likely pathogenic (PM2, PM4, PP1, PP4) |
|  | F366 | 10246 | M | ADPKD | 49  57  57 | Yes | *PKD1*  (AD; Monoallelic) | Het. | Splicing | c.11411+1G>C | 0 | Pathogenic  PVS1, PM2, PP1, PP4 |
|  | F366 | 10247 | M | ADPKD | 28  x  x | Yes | *PKD1*  (AD; Monoallelic) | Het. | Splicing | c.11411+1G>C | 0 | Pathogenic  PVS1, PM2, PP1, PP4 |
|  | F380 | 10212 | M | ADPKD | 21  54  54 | Yes | *PKD1*  *(AD*; Monoallelic*)* | Het. | Frameshift Deletion | c.4782delT  p.Pro1594fs | 0 | Pathogenic  PVS1, PM2, PP1, PP4 |
|  | F380 | 10252 | F | ADPKD | 17  x  x | Yes | *PKD1*  (AD; Monoallelic) | Het. | Frameshift Deletion | c.4782delT  p.Pro1594fs | 0 | Pathogenic  PVS1, PM2, PP1, PP4 |
|  | F443 | 10311 | F | ADPKD | 21  41  41 | Yes | *PKD1*  (AD; Monoallelic) | Het. | Stopgain | c.8056C>T  p.Gln2686Ter | 0 | Pathogenic  PVS1, PM2, PP1, PP4,PP5 |
|  | F443 | 10340 | M | ADPKD | 44  50  52 | Yes | *PKD1*  (AD; Monoallelic) | Het. | Stopgain | c.8056C>T  p.Gln2686Ter | 0 | Pathogenic  PVS1, PM2, PP1,PP4, PP5 |
|  | F115 | 10249 | F | ADPKD | 47  61  66 | Yes | *PKD1*  (AD; Monoallelic) | Het. | Non-synonymous SNV | c.11249G>A  p.Arg3750Gln | 0.000004044 | Likely pathogenic  PM5, PP1, PP3, PP4, PP5 |
|  | F115 | 10336 | M | ADPKD | 55  60  62 | Yes | *PKD1*  (AD; Monoallelic) | Het. | Non-synonymous SNV | c.11249G>A  p.Arg3750Gln | 0.000004044 | Likely pathogenic  PM5, PP1, PP3, PP4, PP5 |
|  | F8 | 8169 | M | ADPKD | 32  38  40 | Yes | *PKD1*  (AD; Monoallelic) | Het. | Stopgain | c.6493C>T  p.Gln2165Ter | 0 | Pathogenic  PVS1, PM2, PP1, PP4 |
|  | F8 | 10142 | F | ADPKD | 44  49  51 | Yes | *PKD1*  (AD; Monoallelic) | Het. | Stopgain | c.6493C>T  p.Gln2165Ter | 0 | Pathogenic  PVS1, PM2, PP1, PP4 |
|  | F685 | 10448 | F | ADPKD | 21  x  x  CKD G1 | Yes | *PKD1*  (AD; Monoallelic) | Het. | Frameshift Deletion | c.2085delC  p.Pro695fs | 0 | Pathogenic  PVS1, PM1, PM2, PP1, PP4 |
|  | F685 | 10473 | M | ADPKD | 21  49  51 | Yes | *PKD1*  (AD; Monoallelic) | Het. | Frameshift Deletion | c.2085delC  p.Pro695fs | 0 | Pathogenic  PVS1, PM1, PM2, PP1, PP4 |
|  | F376 | 10373 | F | ADPKD | N/A  N/A  N/A | Yes | *PKD1*  (AD; Monoallelic) | Het. | Frameshift Insertion | c.11312_11313insTC  p.Ser3771fs | 0 | Pathogenic  PVS1, PM2, PP1, PP4 |
|  | F376 | 10217 | M | ADPKD | 44  46  47 | Yes | *PKD1*  (AD; Monoallelic) | Het. | Frameshift Insertion | c.11312_11313insTC  p.Ser3771fs | 0 | Pathogenic  PVS1, PM2, PP1, PP4 |
|  | F376 | 10440 | M | ADPKD | 3  Pre-emptive KTx  35 | Yes | *PKD1*  (AD; Monoallelic) | Het. | Frameshift Insertion | c.11312_11313insTC  p.Ser3771fs | 0 | Pathogenic  PVS1, PM2, PP1, PP4 |
|  | F376 | 10286 | F | ADPKD | 21  41  44 | Yes | *PKD1*  (AD; Monoallelic) | Het. | Frameshift Insertion | c.11312_11313insTC  p.Ser3771fs | 0 | Pathogenic  PVS1, PM2, PP1, PP4 |
|  | F376 | 10208 | F | ADPKD | 15  36  39 | Yes | *PKD1*  (AD; Monoallelic) | Het. | Frameshift Insertion | c.11312_11313insTC  p.Ser3771fs | 0 | Pathogenic  PVS1, PM2, PP1, PP4 |
|  | F631 | 10639 | M | ADPKD | 34  64  x | Yes | *PKD2*  (AD; Monoallelic) | Het. | Stopgain | c.1480G>T  p.Glu494Ter | 0 | Pathogenic  PVS1, PM2, PP1, PP3, PP4, PP5 |
|  | F631 | 10643 | F | ADPKD | 49  x  x | Yes | *PKD2*  (AD; Monoallelic) | Het. | Stopgain | c.1480G>T  p.Glu494Ter | 0 | Pathogenic  PVS1, PM2, PP1, PP3, PP4, PP5 |
|  | F631 | 10646 | M | ADPKD | 45  x  x | Yes | *PKD2*  (AD; Monoallelic) | Het. | Stopgain | c.1480G>T  p.Glu494Ter | 0 | Pathogenic  PVS1, PM2, PP1, PP3, PP4, PP5 |
|  | F631 | 10640 | F | ADPKD | 58  x  x | Yes | *PKD2*  (AD; Monoallelic) | Het. | Stopgain | c.1480G>T  p.Glu494Ter | 0 | Pathogenic  PVS1, PM2, PP1, PP3, PP4, PP5 |
|  | F631 | 10641 | F | ADPKD | 42  x  x | Yes | *PKD2*  (AD; Monoallelic) | Het. | Stopgain | c.1480G>T  p.Glu494Ter | 0 | Pathogenic  PVS1, PM2, PP1, PP3, PP4, PP5 |
|  | F393 | 10326 | F | ADPKD | 48  57  Pre-emptive Tx | Yes | *PKD1*  (AD; Monoallelic) | Het | Splice region  (Intronic) | c.11017-10C>A | 0 | Likely Pathogenic PM2, PP3, PP4, PP5 |
|  | F393 | 11225 | F | ADPKD | 29  x  x  CKD G1 | Yes | *PKD1*  (AD; Monoallelic) | Het | Splice region  (Intronic) | c.11017-10C>A | 0 | Likely Pathogenic PM2, PP3, PP4, PP5 |
|  | F821 | 10878 | F | ADPKD | 56  x  x  CKD G2 | Yes | *PKD1*  (AD; Monoallelic) | Het | Frameshift Insertion | c.7000_7001insGCTGGCG  p.Val2334fs | 0 | Pathogenic PVS1, PM2, PP4, PP5 |
|  | F821 | 11016 | M | ADPKD | 23  x  x  CKD G1 | Yes | *PKD1*  (AD; Monoallelic) | Het | Frameshift Insertion | c.7000_7001insGCTGGCG  p.Val2334fs | 0 | Pathogenic PVS1, PM2, PP4, PP5 |
|  | F674 | 8162 | F | ADPKD | 23  x  x  CKD G4 | Yes | *PKD1*  (AD; Monoallelic) | Het. | Stopgain | c.12673C>T  p.Gln4225Ter | 0 | Pathogenic  PVS1, PM2, PP3, PP4,PP5 |
|  | F674 | 8194 | F | ADPKD | 35  63  63 | Yes | *PKD1*  (AD; Monoallelic) | Het. | Stopgain | c.12673C>T  p.Gln4225Ter | 0 | Pathogenic  PVS1, PM2, PP3, P4,PP5 |
|  | F461 | 10644 | F | ADPKD | 57  x  x  CKD G2 | Yes | *PKD2*  (AD; Monoallelic) | Het | Frameshift Insertion | c.195_196insGACCp.Arg65fs | 0 | Pathogenic  PVS1, PM2, PP1, PP4 |
|  | F461 | 10018 | M | ADPKD | 35  54  x | Yes | *PKD2*  (AD; Monoallelic) | Het | Frameshift Insertion | c.195_196insGACCp.Arg65fs | 0 | Pathogenic  PVS1, PM2, PP1, PP4 |
|  | F461 | 10345 | F | ADPKD | 47  x  x | Yes | *PKD2*  (AD; Monoallelic) | Het | Frameshift Insertion | c.195_196insGACCp.Arg65fs | 0 | Pathogenic  PVS1, PM2, PP1, PP4 |
|  | F20 | 8128 | M | ADPKD | 27  67  69 | Yes | *PKD1*  (AD; Monoallelic) | Het. | Non-synonymous SNV | c.11249G>A  p.Arg3750Gln | 0.000004044 | Likely pathogenic  PM5, PP1, PP3, PP4, PP5 |
|  | F20 | 10213 | F | ADPKD | 16  44  48 | Yes | *PKD1*  (AD; Monoallelic) | Het. | Non-synonymous SNV | c.11249G>A  p.Arg3750Gln | 0.000004044 | Likely pathogenic  PM5, PP1, PP3, PP4, PP5 |
|  | F354 | 10271 | M | ADPKD | 65  75  x | Yes | *PKD2*  (AD; Monoallelic) | Het. | Stopgain | c.2286C>A  p.Tyr762Ter | 0 | Pathogenic  PVS1, PM2, PP4, PP5 |
|  | F354 | 10371 | F | ADPKD | N/A  N/A  N/A | Yes | *PKD2*  (AD; Monoallelic) | Het. | Stopgain | c.2286C>A  p.Tyr762Ter | 0 | Pathogenic  PVS1, PM2, PP4, PP5 |
|  | F360 | 10358 | M | ADPKD | 40  55  57 | Yes | *PKD1*  (AD; Monoallelic) | Het. | Frameshift Deletion | c.5142delC  p.Phe1714fs | 0 | Pathogenic  PVS1, PM2, PP1, PP4, PP5 |
|  | F360 | 10495 | F | ADPKD | 19  x  x  CKD G1 | Yes | *PKD1*  (AD; Monoallelic) | Het. | Frameshift Deletion | c.5142delC  p.Phe1714fs | 0 | Pathogenic  PVS1, PM2, PP1, PP4, PP5 |
|  | F769 | 10337 | M | ADPKD | 45  x  x  CKD G3 | Yes | *PKD1*  (AD; Monoallelic) | Het. | Non-frameshift Deletion | c.3719_3721del  p.1240_1241del | 0 | Likely Pathogenic  PM2, PM4, PP4, PP5 |
|  | F769 | 10943 | M | ADPKD | 48  69  71 | Yes | *PKD1*  (AD; Monoallelic) | Het. | Non-Frameshift Deletion | c.3719_3721del  p.1240_1241del | 0 | Likely pathogenic PM2, PM4, PP4, PP5 |
|  | F19 | 11084 | M | ADPKD | 29  47  49 | Yes | *PKD1*  (AD; Monoallelic) | Het | Splicing | c.288-2A>G | 0 | Pathogenic PVS1, PM2, PP4, PP5 |
|  | F401 | 11156 | F | ADPKD | 43  53  54 | Yes | *PKD1*  (AD; Monoallelic) | Het. | Frameshift Insertion | c.11699_11700insCTCTCGCTGCC  p.Pro3900fs | 0 | Pathogenic PVS1, PM2, PP4 |
|  | F329 | 8124 | M | ADPKD | 46  49  50 | No | *PKD2*  (AD; Monoallelic) | Het. | Non-synonymous SNV | c.964C>T  p.Arg322Trp | 0 | Likely pathogenic  PM2, PP2, PP3, PP4, PP5 |
|  | F60 | 8127 | F | ADPKD | 25  45  49 | Yes | *PKD1*  (AD; Monoallelic) | Het. | Stopgain | c.8056C>T  p.Gln2686Ter | 0 | Pathogenic  PVS1, PM2, PP1, PP4,PP5 |
|  | F427 | 8137 | F | ADPKD | 34  49  x | Yes | *PKD1*  (AD; Monoallelic) | Het. | Frameshift Insertion | c.439dupG  p.Val147fs | 0 | Pathogenic  PVS1, PM2, PP4 |
|  | F428 | 8141 | F | ADPKD | 51  x  x  CKD G1 | Yes | *PKD2*  (AD; Monoallelic) | Het. | Non-frame shift Deletion | c.982_984del  p.328_328del | 0 | Likely pathogenic  PM2, PM4, PP2, PP4 |
|  | F429 | 8142 | F | ADPKD | 21  x  x  CKD G3a | Yes | *PKD2*  (AD; Monoallelic) | Het. | Frameshift Deletion | c.1551delG  p.Leu517fs | 0.000004089 | Pathogenic  PVS1, PP4, PP5 |
|  | F438 | 8145 | F | ADPKD | 25  x  x  CKD G2 | Yes | *PKD1*  (AD; Monoallelic) | Het. | Frameshift Deletion | c.5014_5015del  p.Arg1672fs | 0 | Pathogenic  PVS1, PM2, PP4 |
|  | F436 | 8151 | M | ADPKD | 63  63  70 | Yes | *PKD2*  (AD; Monoallelic) | Het. | Splicing | c.1094+1G>A | 0 | Pathogenic  PVS1, PM2, PP3, PP4, PP5 |
|  | F624 | 8167 | F | ADPKD | 30  x  x | Yes | *PKD1*  *(AD*; Monoallelic*)* | Het. | Stopgain | c.12682C>T  p.Arg4228Ter | 0.000004083 | Pathogenic  PVS1, PM1, PP4, PP5 |
|  | F641 | 8179 | M | ADPKD | 19  40  42 | Yes | *PKD1*  (AD; Monoallelic) | Het. | Stopgain | c.7903G>T  p.Glu2635Ter | 0 | Pathogenic  PVS1, PM2, PP4 |
|  | F394 | 8181 | F | ADPKD | 19  34  34 | Yes | *PKD1*  (AD; Monoallelic) | Het. | Stopgain | c.6367C>T  p.Gln2123Ter | 0 | Pathogenic  PVS1, PM2, PP4 |
|  | F637 | 8182 | F | ADPKD | 40  57  62 | No | *PKD1*  (AD; Monoallelic) | Het. | Frameshift Deletion | c.5014_5015del  p.Arg1672fs | 0 | Pathogenic  PVS1, PM2, PP4 |
|  | F5 | 8192 | F | ADPKD | 20  46  48 | Yes | *PKD1*  (AD; Monoallelic) | Het. | Frameshift Deletion | c.12604_12631del  p.Gly4202fs | 0 | Pathogenic  PVS1, PM2, PP4, PP5 |
|  | F675 | 8193 | F | ADPKD | 38  40  40 | No | *PKD1*  (AD; Monoallelic) | Het. | Frameshift Deletion | c.711delC  p.Pro237fs | 0 | Pathogenic  PVS1, PM2,PP4 |
|  | F331 | 10019 | F | ADPKD | 35  66  67 | Yes | *PKD2*  (AD; Monoallelic) | Het. | Splicing | c.2020-2A>- | 0 | Pathogenic  PVS1, PM2, PP4 |
|  | F332 | 10020 | F | ADPKD | 24  37  42 | Yes | *PKD1*  (AD; Monoallelic) | Het. | Frameshift Deletion | c.9736delC  p.Arg3246fs | 0 | Pathogenic  PVS1, PM2, PP4 |
|  | F333 | 10021 | M | ADPKD | 40  52  x | Yes | *PKD1*  (AD; Monoallelic) | Het. | Stopgain | c.11525G<A  p.Trp3842Ter | 0 | Pathogenic  PVS1, PM2, PP3, PP4, PP5 |
|  | F79 | 10022 | M | ADPKD | 26  44  45 | Yes | *PKD1*  (AD; Monoallelic) | Het. | Stopgain | c.3349C>T  p.Gln1117Ter | 0 | Pathogenic  PVS1, PM2, PP4, PP5 |
|  | F337 | 10139 | M | ADPKD | 23  43  43 | No | *PKD1*  (AD; Monoallelic) | Het. | Non-synonymous SNV | c.11614G>C  p.Glu3872Gln | 0 | VUS  PM2, PP3, PP4, PP5  Highly Likely Pathogenic |
|  | F285 | 10141 | F | ADPKD | 40  43  47 | Yes | *PKD1*  (AD; Monoallelic) | Het. | Stopgain | c.6846C>A  p.Tyr2282Ter | 0 | Pathogenic  PVS1, PM2, PP4, PP5 |
|  | F339 | 10200 | M | ADPKD | 18  25  26 | Yes | *PKD1*  (AD; Monoallelic) | Het. | Frameshift Deletion | c.7495delC  p.His2499fs | 0 | Pathogenic PVS1, PM1, PM2, PP4 |
|  | F340 | 10201 | M | ADPKD | 55  66  x | No | *PKD1*  (AD; Monoallelic) | Het. | Stopgain | c.11752G>T  p.Glu3918Ter | 0 | Pathogenic  PVS1, PM2, PP4, PP5 |
|  | F341 | 10202 | M | ADPKD | 38  62  64 | Yes | *PKD1*  (AD; Monoallelic) | Het. | Stopgain | c.2534T>A  p.Ler845Ter | 0 | Pathogenic  PVS1, PM2, PP3, PP4 |
|  | F638 | 8175 | F | ADPKD | 44  47  50 | No | *PKD1*  (AD; Monoallelic) | Het | Splicing | c.12139-2A>C | 0 | Pathogenic  PVS1, PM1,PP4 |
|  | F378 | 10209 | F | ADPKD | 50  x  x | Yes | *PKD2*  (AD; Monoallelic) | Het. | Stopgain | c.602G>A  p.Trp201Ter | 0 | Pathogenic  PVS1, PM2, PP3, PP4, PP5 |
|  | F151 | 10214 | F | ADPKD | 45  47  49 | Yes | *PKD1*  (AD; Monoallelic*)* | Het. | Non-frameshift Deletion | c.7303_7317del  p.2435_2439del | 0 | Likely pathogenic  PM2, PM4, PP3, PP4 |
|  | F353 | 10216 | M | ADPKD | 29  45  46 | Yes | *PKD1*  (AD; Monoallelic) | Het. | Stopgain | c.12673C>T  p.Gln4225Ter | 0 | Pathogenic  PVS1, PM2, PP3, P4,PP5 |
|  | F371 | 10218 | M | PKD | 51  62  66 | No | *PKD1*  (AD; Monoallelic) | Het. | Stopgain | c.99C>A  p.Cys33Ter | 0 | Pathogenic  PVS1, PM2, PP4 |
|  | F345 | 10219 | F | ADPKD | 24  30  31 | Yes | *PKD1*  (AD; Monoallelic) | Het. | Stopgain | c.6199C>T  p.Gln2067Ter | 0 | Pathogenic  PVS1, PM2, PP4, PP5 |
|  | F385 | 10221 | M | ADPKD | 27  59  61 | Yes | *PKD1*  (AD; Monoallelic) | Het. | Stopgain | c.7369G>T  p.Glu2457Ter | 0 | Pathogenic  PVS1, PM2, PP4 |
|  | F357 | 10225 | F | ADPKD | 27  43  53 | Yes | *PKD1*  (AD; Monoallelic) | Het. | Frameshift Insertion | c.1171dupA  p.Ile391fs | 0 | Pathogenic  PVS1, PM2, PP4 |
|  | F383 | 10245 | F | ADPKD | 60  62  63 | No | *PKD1*  (AD; Monoallelic) | Het. | Stopgain | c.7833C>G  p.Tyr2611Ter | 0.00000437 | Pathogenic  PVS1, PM2, PP4 |
|  | F346 | 10261 | M | ADPKD | 42  42  43 | No | *PKD1*  (AD; Monoallelic) | Het. | Splicing | c.2986-1G>A | 0 | Pathogenic (Ic)  PVS1, PM2, PP4 |
|  | F370 | 10280 | M | ADPKD | 45  67  69 | Yes | *PKD1*  (AD; Monoallelic) | Het. | Frameshift Deletion | c.348_352del  p.Asn116fs | 0 | Pathogenic  PVS1, PM2 |
|  | F375 | 10282 | M | PKD | 42  46  48 | No | *PKD1*  (AD; Monoallelic) | Het. | Frameshift Deletion | c.4654delG  p.Val1552fs | 0 | Pathogenic  PVS1, PM2, PP4 |
|  | F408 | 10287 | M | PKD | N/A  N/A  N/A | N/A | *PKD1*  (AD; Monoallelic) | Het. | Non-synonymous SNV | c.8299C>T  p.Arg2767Cys | 0 | Likely pathogenic  PM1, PM2, PP3, PP4, PP5 |
|  | F367 | 10288 | F | ADPKD | 37  45  46 | Yes | *PKD1*  (AD; Monoallelic) | Het. | Frameshift Deletion | c.11454delC  p.Gly3818fs | 0 | Pathogenic  PVS1, PM2, PP4 |
|  | F453 | 10290 | M | ADPKD | 20  x  x | Yes | PKD1  (AD; Monoallelic) | Het. | Splicing | c.2097+2T>C | 0 | Pathogenic  PVS1, PM2, PP3 |
|  | F300 | 10296 | M | ADPKD | 47  47  48 | Yes | *PKD1*  (AD; Monoallelic) | Het. | Stopgain | c.1303CT  p.Gln435Ter | 0 | Pathogenic  PVS1, PM2, PP4 |
|  | F439 | 10308 | F | ADPKD | 44  54  66 | Yes | *PKD1*  (AD; Monoallelic) | Het. | Splicing | c.1606+5G>A | 0 | Pathogenic  PVS1, PM2, PP4 |
|  | F441 | 10309 | M | PKD | 40  58  60 | No | PKD1  (AD; Monoallelic) | Het. | Stopgain | c.8008C>T  p.Gln2670Ter | 0 | Pathogenic  PVS1, PM2, PP3, PP4, PP5 |
|  | F369 | 10315 | F | ADPKD | 26  48  54 | Yes | *PKD1*  (AD; Monoallelic) | Het. | Splicing | c.6919-9G>A | 0 | Pathogenic  PVS1, PP3, PP4 |
|  | F463 | 10338 | F | ADPKD | 35  x  x | Yes | *PKD1*  (AD; Monoallelic) | Het. | Frameshift Insertion | c.11395_11396insGGGACGTGGGCCTATTCAG  p.Ala3799fs | 0 | Pathogenic  PVS1, PM2, PP4 |
|  | F466 | 10341 | F | ADPKD | 15  49  50 | Yes | *PKD1*  (AD; Monoallelic) | Het. | Frameshift Deletion | c.2032delG  p.Ala678fs | 0 | Pathogenic  PVS1, PM2, PP4 |
|  | F363 | 10357 | M | ADPKD | 64  68  75 | Yes | *PKD2*  (AD; Monoallelic) | Het. | Stopgain | c.602G>A  p.Trp201Ter | 0 | Pathogenic  PVS1, PM2, PP3, PP4, PP5 |
|  | F28 | 10359 | M | ADPKD | 25  50  x | Yes | *PKD1*  (AD; Monoallelic) | Het. | Splicing | c.288-2A>G | 0 | Pathogenic  PVS1, PM2, PP4, PP5 |
|  | F603 | 10381 | M | ADPKD | 63  x  x | No | *PKD1*  (AD; Monoallelic) | Het. | Non-synonymous SNV | c.10942C>A  p.Pro3648Thr | 0 | Likely pathogeni  PM2, PM5, PP3, PP4 |
|  | F476 | 10392 | M | ADPKD | 45  50  51 | No | *PKD1*  (AD; Monoallelic) | Het. | Splicing | c.8017-1G>C | 0 | Pathogenic  PVS1, PM2, PP4 |
|  | F680 | 10430 | M | ADPKD | 53  57  59 | No | *PKD1*  (AD; Monoallelic) | Het. | CNV | - | 0 | CNV  Large deletion in key gene |
|  | F342 | 10443 | M | ADPKD | 12  x  x | Yes | *PKD1*  (AD; Monoallelic) | Het. | Stopgain | c.7987C>T  p.Gln2663Ter | 0 | Pathogenic  PVS1, PM2, PP4, PP5 |
|  | F690 | 10467 | F | ADPKD | 21  41  41 | Yes | *PKD1*  (AD; Monoallelic) | Het. | Frameshift Deletion | c.11563_11564del  p.Thr3855fs | 0 | Pathogenic  PVS1, PM2, PP4, PP5 |
|  | F687 | 10471 | F | ADPKD | 25  x  x | Yes | *PKD1*  (AD; Monoallelic) | Het. | Non-synonymous SNV | c.2534T>C  p.Leu845Ser | 0.0000043 | Likely pathogenic  PS1, PP3, PP4 |
|  | F688 | 10472 | F | ADPKD | 13  46  46 | Yes | *PKD1*  (AD; Monoallelic) | Het. | CNV | 5114 bp deletion | 0 | CNV  Large deletion in key gene |
|  | F698 | 10481 | F | ADPKD | 28  53  x | Yes | *PKD1*  (AD; Monoallelic) | Het. | CNV | 19770 bp deletion | 0 | CNV  Large deletion in key gene |
|  | F616 | 10627 | M | ADPKD | 35  47  x | Yes | *PKD1*  (AD; Monoallelic) | Het. | Frameshift Insertion | c.11984dupT  p.Ler3995fs | 0 | Pathogenic  PVS1, PM2, PP4 |
|  | F617 | 10628 | F | ADPKD | 38  48  x | Yes | *PKD1*  (AD; Monoallelic*)* | Het. | Frameshift Deletion | c.6727_6728del  p.Gln2243fs | 0 | Pathogenic  PVS1, PM2, PP4, PP5 |
|  | F623 | 10634 | F | ADPKD | 54  61  x | Yes | *PKD1*  (AD; Monoallelic) | Het. | Stopgain | c.11343C>G  p.Tyr3781Ter | 0 | Pathogenic  PVS1, PM2, PP1, PP4, PP5 |
|  | F627 | 10636 | F | ADPKD | 60  61  64 | Yes | *PKD1*  (AD; Monoallelic) | Het. | Frameshift Insertion | c.2102_2103insAC  p.(Thr701fs) | 0 | Pathogenic  PVS1, PM2, PP4 |
|  | F629? | 10638 | F | ADPKD | 11  x  x | Yes | *PKD1*  (AD; Monoallelic) | Het. | Non-frameshift Deletion | c.8284_8295del  p.2762_2765del | 0 | Likely pathogenic  PM2, PM4, PP4, PP5 |
|  | F644 | 10647 | F | ADPKD | 58  x  x | Yes | *PKD2*  (AD; Monoallelic) | Het. | Stopgain | c.2614C>T  p.Arg872Ter | 0 | Pathogenic  PVS1, PM2, PP3, PP4, PP5 |
|  | F645 | 10648 | M | ADPKD | 39  55  x | Yes | *PKD1*  (AD; Monoallelic) | Het. | Frameshift Deletion | c.2396_2421del  p.Arg799fs | 0 | Pathogenic  PVS1, PM2, PP4 |
|  | F647 | 10650 | F | ADPKD | 31  56  56 | Yes | *PKD1*  (AD; Monoallelic) | Het. | Stopgain | c.3184C>T  p.Gln1062Ter | 0 | Pathogenic  PVS1, PM2, PP4, PP5 |
|  | F531 | 10652 | F | ADPKD | 22  48  53 | Yes | *PKD1*  (AD; Monoallelic) | Het. | Frameshift Insertion | c.12039_12040insAC  p.Val4014fs | 0 | Pathogenic  PVS1, PM2, PP4 |
|  | F498 | 10653 | M | ADPKD | 61  71  x | No | *PKD2*  (AD; Monoallelic) | Het. | Non-synonymous SNV | c.964C>T  p.Arg322Trp | 0 | Likely pathogenic  PM2, PP2, PP3, PP4, PP5 |
|  | F650 | 10655 | F | ADPKD | 46  69  x | Yes | *PKD1*  (AD; Monoallelic) | Het. | Stopgain | c.8008C>T  p.Gln2670Ter | 0 | Pathogenic  PVS1, PM2, PP3, PP4, PP5 |
|  | F651 | 10656 | M | ADPKD | 38  x  x | Yes | *PKD1*  (AD; Monoallelic) | Het. | Non-frameshift Deletion | c.8935_8937del  p.2979_2979del | 0 | Likely pathogenic  PM2, PM4, PP4, PP5 |
|  | F652 | 10657 | F | ADPKD | 62  62  67 | Yes | *PKD2*  (AD; Monoallelic) | Het. | Non-synonymous SNV | c.974G>A  p.Arg325Gln | 0 | Likely pathogenic  PM2, PP2, PP3, PP4, PP5 |
|  | F653 | 10658 | M | ADPKD | 43  x  x | Yes | *PKD1*  (AD; Monoallelic) | Het. | Non-frameshift Deletion | c.3697_3699delGCC  p.A1233del | 0 | Likely pathogenic  PM1, PM2, PM4, PP4 |
|  | F657 | 10660 | M | PKD | 40  x  x | No | *PKD1*  (AD; Monoallelic) | Het. | Frameshift Insertion | c.4232_4233insTTCCCCTACCG  p.Arg1411fs | 0 | Pathogenic  PVS1, PM2, PP4 |
|  | F658 | 10661 | F | ADPKD | 26  55  55 | Yes | *PKD1*  (AD; Monoallelic) | Het. | Frameshift Deletion | c.4452delG  p.Pro1484fs | 0 | Pathogenic  PVS1, PM2, PP4 |
|  | F660 | 10662 | F | PKD | 52  x  x | No | *PKD2*  (AD; Monoallelic) | Het. | Non-synonymous SNV | c.964C>T  p.Arg322Trp | 0 | Likely pathogenic  PM2, PP2, PP3, PP4, PP5 |
|  | F661 | 10663 | M | ADPKD | 38  58  58 | Yes | *PKD2*  (AD; Monoallelic) | Het. | Frameshift Deletion | c.2376_2382del  p.Asp792fs | 0 | Pathogenic  PVS1, PM2, PP4 |
|  | F491 | 10684 | F | ADPKD | 23  x  x | Yes | *PKD2*  (AD; Monoallelic) | Het. | Stopgain | c.744C>A  p.Tyr248Ter | 0 | Pathogenic  PVS1, PM2, PP4 |
|  | F672 | 10686 | F | ADPKD | 23  x  x | Yes | *PKD2*  (AD; Monoallelic) | Het. | Non-synonymous SNV | c.964C>T  p.Arg322Trp | 0 | Likely pathogenic  PM2, PP2, PP3, PP4, PP5 |
|  | F754 | 10921 | F | PKD | 33  x  x | No | *PKD1*  (AD; Monoallelic) | Het. | Frameshift Deletion | c.3684delC  p.Pro1228fs | 0 | Pathogenic  PVS1, PM2, PP4, PP5 |
|  | F771 | 10961 | F | PKD | 22  x  x | No | *PKD1*  (AD; Monoallelic) | Het. | Non-synonymous SNV | c.7215G>T  p.Trp2405Cys | 0 | Likely pathogenic  PM2, PM5, PP3, PP4 |
|  | F709 | 28115 | F | ADPKD | 22  x  x | Yes | *PKD1*  (AD; Monoallelic) | Het. | Frameshift Insertion | c.8234_8235insCGTCACCATC  p.Ser2745fs | 0 | Pathogenic  PVS1, PM2, PP4 |
|  | F726 | 28153 | M | ADPKD | 39  x  x | Yes | *PKD2*  (AD; Monoallelic) | Het. | Frameshift Deletion | c.405delC  p.Gly135fs | 0 | Pathogenic  PVS1, PM2, PP4 |
|  | F361 | 10266 | M | ADPKD | 32  37  38 | Yes | *PKD1*  (AD; Monoallelic) | Het. | Splicing | c.11538-2A>T | 0 | Pathogenic PVS1, PM2, PP4, PP5 |
|  | F1108 | 10811 | F | ADPKD | 58  57  x | Yes | *PKD1*  (AD; Monoallelic) | Het. | Frameshift Deletion | c.2396_2421del  p.Arg799fs | 0 | Pathogenic PVS1, PM2, PP4 |
|  | F396 | 10257 | M | ADPKD | 23  x  x | No | *PKD1*  (AD; Monoallelic) | Het. | Frameshift Insertion | c.11713dupG  p.Val3905Glyfs | 0 | Pathogenic PVS1, PM2, PP4 |
|  | F734 | 28177 | F | ADPKD | 25  x  x  CKD G2 | Yes | *PKD1*  (AD; Monoallelic) | Het. | Splicing | c.3296-2A>G | 0 | Likely Pathogenic PVS1, PM2 |
|  | F745 | 10985 | M | ADPKD | 43  x  x | Yes | *PKD1*  (AD; Monoallelic) | Het. | Splicing | c.288-2A>G | 0 | Pathogenic PVS1, PM2, PP4, PP5 |
|  | F787 | 10952 | F | ADPKD | 10  x  x  CKD G1 | Yes | *PKD1*  (AD; Monoallelic) | Het. | Non-synonymous SNV | c.12448C>T  p.Arg4150Cys | 0 | Likely pathogenic PS1, PM2, PP4 |
|  | F817 | 11006 | F | ADPKD | 39  x  x  CKD G1 | Yes | *PKD1*  (AD; Monoallelic) | Het. | Splicing | c.1607-2A>G | 0 | Pathogenic PVS1, PM2 |
|  | F1048 | 11090 | M | ADPKD | 26  x  x  CKD G2 | Yes | *PKD1*  (AD; Monoallelic) | Het. | Frameshift Insertion | c.9388dupC  p.Arg3130fs | 0 | Pathogenic PVS1, PM2, PP4, PP5 |
|  | F957 | 11221 | M | ADPKD | 27  x  x  CKD G1 | Yes | *PKD1*  (AD; Monoallelic) | Het. | Stopgain | c.4306C>T  p.Arg1436Ter | 0 | ACMG Pathogenic |
|  | F967 | 10861 | M | ADPKD | 26  48  50 | Yes | *PKD1*  (AD; Monoallelic) | Het. | Frameshift Insertion | c.5014_5015del  p.ARg1672fs | 0 | Pathogenic PVS1, PM2, PP4, PP5 |
|  | F646 | 10666 | M | ADPKD | 45  x  x | Yes | *PKD2*  (AD; Monoallelic) | Het. | Stopgain | c.1480G>T  p.Glu494Ter | 0 | Pathogenic  PVS1, PM2, PP1, PP3, PP4, PP5 |
|  | TF88 | 11101 | F | ADPKD | 26  56  74 | Yes | *PKD1*  (AD; Monoallelic) | Het. | Stopgain | c.430C>T  p.Gln144Ter | 0 | Pathogenic PVS1, PM2,PP4 |
|  | F67 | 10379 | M | ADPKD | 12  48  x | Yes | *PKD1*  (AD; Monoallelic) | Het. | Stopgain | c.11119C>T  p.Gln3707Ter | 0 | Pathogenic  PVS1, PM2, PP4, PP5 |
|  | F170 | 8117 | F | ADPKD | 20  39  40 | Yes | *PKD1*  (AD; Monoallelic) | Het. | Stopgain | c.11343C>G  p.Tyr3781Ter | 0 | Pathogenic  PVS1, PM2, PP4, PP5 |
|  | F327 | 8116 | M | ADPKD | 27  43  45 | Yes | *PKD1*  (AD; Monoallelic) | Het. | Stopgain | c.8045C>A  p.Ser2682Ter | 0 | Pathogenic  PVS1, PM2, PP4, PP5 |
|  | F334 | 10023 | M | ADPKD | 13  x  x  CKD G1 | Yes | *PKD1*  (AD; Monoallelic) | Het. | Frameshift Deletion | c.1004_1019del  p.Ala335fs | 0 | Pathogenic  PVS1, PM2, PP4 |
|  | F573 | 28149 | M | ADPKD | 40  57  60 | Yes | *PKD2*  (AD; Monoallelic) | Het. | Frameshift Deletion | c.1551delG  p.Leu517fs | 0 | Pathogenic  PVS1, PM2, PP4, PP5 |
|  | F571 | 10494 | M | ADPKD | 27  Pre-emptive KTx  42 | Yes | *PKD1*  (AD; Monoallelic) | Het. | Non-frameshift Deletion | c.9859_9861del  p.3287_3287del | 0 | Likely pathogenic  PM2, PM4, PP4, PP5 |
|  | F633 | 10642 | F | ADPKD | 45  x  x | Yes | *PKD2*  (AD; Monoallelic) | Het. | Stopgain | c.2614C>T  p.Arg872Ter | 0 | Pathogenic  PVS1, PP4, PP5 |
|  | F400 | 10265 | F | PKD | 21  X  X  CKD G1 | No | *PKD1*  (AD; Monoallelic) | Het. | Splicing | c.1606+5G>A | 0 | Pathogenic  PVS1, PM2, PP4 |
|  | F234 | 28185 | M | ADPKD | x  53  54 | Yes | *PKD1*  (AD) | Het. | Non-synonymous SNV - LR-PCR | - | - | - |
|  | F157 | 28157 | M | ADPKD | 30  40  41 | Yes | *PKD1*  (AD; Monoallelic) | Het. | Frameshift Deletion | c.78_delC  p.Arg28fs | 0 | Pathogenic  PVS1, PM2, PP5 |
|  | F729 | 10902 | M | ADPKD | 54  56  62 | Yes | *PKD1*  (AD) | Het. | Non-synonymous SNV - LR-PCR | - | - | - |
|  | F813 | 28137 | F | ADPKD | 34  x  x | Yes | *PKD1*  (AD) | Het. | Non-synonymous SNV - LR-PCR | - | - | - |
|  | F782 | 10979 | F | ADPKD | 16  x  x  CKD G1 | Yes | *PKD1*  (AD; Monoallelic) | Het. | Non-synonymous SNV | c.1831C>T  p.Arg611Trp | 0 | VUS  PM2, PP3, PP4, PP5  (considered disease-causing) |
|  | F782 | 10236 | F | ADPKD | 50  x  x  CKD G4 | Yes | *PKD1*  (AD; Monoallelic) | Het. | Non-synonymous SNV | c.1831C>T  p.Arg611Trp | 0 | VUS  PM2, PP3, PP4, PP5 (considered disease-causing) |
|  | F773 | 8200 | M | ADPKD | 23  29  31 | Yes | *PKD1*  (AD; Monoallelic) | Het. | Non-synonymous SNV | c.9425T>C  p.Leu3142Pro | 0 | VUS  PM2,PP3, PP4, PP5 (considered disease-causing) |
|  | F773 | 11149 | F | ADPKD | 28  57  61 | Yes | *PKD1*  (AD; Monoallelic) | Het. | Non-synonymous SNV | c.9425T>C  p.Leu3142Pro | 0 | VUS  PM2,PP3, PP4, PP5 (considered disease-causing) |
|  | F368 | 10025 | F | ADPKD | 30  32  33 | Yes | *PKD1*  (AD; Monoallelic) | Het. | Non-synonymous SNV | c.4082T>C  p.Leu1361Pro | 0 | VUS  PM2, PP4, PP5 (considered disease-causing) |
|  | F368 | 10263 | F | ADPKD | 28  60  60 | Yes | *PKD1*  (AD; Monoallelic) | Het. | Non-synonymous SNV | c.4082T>C  p.Leu1361Pro | 0 | VUS  PM2, PP4, PP5 (considered disease-causing) |
|  | F776 | 10950 | M | ADPKD | 7  43  43 | Yes | *PKD1*  (AD; Monoallelic) | Het. | Non-synonymous SNV | c.9143T>G  p.Leu3048Arg | 0 | VUS  PM2, PP1, PP3, PP4 (considered disease-causing) |
|  | F776 | 8146 | M | ADPKD | 32  45  48 | Yes | *PKD1*  (AD; Monoallelic) | Het. | Non-synonymous SNV | c.9143T>G  p.Leu3048Arg | 0 | VUS  PM2, PP1, PP3, PP4 (considered disease-causing) |
|  | F776 | 10272 | F | ADPKD | 17  60  x | Yes | *PKD1*  (AD; Monoallelic) | Het. | Non-synonymous SNV | c.9143T>G  p.Leu3048Arg | 0 | VUS  PM2, PP1, PP3, PP4 (considered disease-causing) |
|  | F528 | 10685 | F | ADPKD | 32  48  50 | Yes | *PKD1*  (AD; Monoallelic) | Het. | Non-synonymous SNV | c.10946C>A  p.Pro3649His | 0 | VUS  PM2, PP3, PP4 (considered disease-causing) |
|  | F615 | 10626 | F | ADPKD | 39  49  53 | Yes | *PKD1*  (AD; Monoallelic) | Het. | Non-synonymous SNV | c.11258G>A  p.Arg3753Gln | 0 | VUS  PM2, PP3, PP4, PP5 (considered disease-causing) |
|  | F686 | 10474 | M | ADPKD | 39  66  67 | Yes | *PKD1*  (AD; Monoallelic) | Het. | Non-synonymous SNV | c.2180T>C  p.Leu727Pro | 0 | VUS  PM2, PP3, PP4, PP5 (considered disease-causing) |
|  | F422 | 10393 | M | PKD | Child  30  57 | No | *PKD1*  (AD; Monoallelic) | Het. | Non-synonymous SNV | c.9425T>C  p.Leu3142Pro | 0 | VUS  PM2,PP3, PP4, PP5 (considered disease-causing) |
|  | F568 | 10378 | F | ADPKD | 30  x  x | Yes | *PKD1*  (AD; Monoallelic) | Het. | Non-synonymous SNV | 3:c.7300C>T  p.Arg2434Trp | 0.000004353 | VUS  PP3, PP4, PP5 (considered disease-causing) |
|  | F607 | 10377 | M | PKD | 27  62  63 | No | *PKD1*  (AD; Monoallelic) | Het. | Non-synonymous SNV | c.9395C>T  p.Ser3132Leu | 0 | VUS  PM2, PP3, PP4, PP5 (considered disease-causing) |
|  | F33 | 10281 | F | ADPKD | 25  48  52 | Yes | *PKD1*  (AD; Monoallelic) | Het. | Non-synonymous SNV | c.301A>G  p.Asn101Asp | 0 | VUS  PM2, PP4, PP5 (considered disease-causing) |
|  | F414 | 10277 | M | PKD | 50  N/A  N/A | No | *PKD2*  (AD; Monoallelic) | Het. | Non-synonymous SNV | c.1345G>C  p.Gly449Arg | 0 | VUS  PM2, PP3, PP4, PP5 (considered disease-causing) |
|  | F348 | 10244 | M | ADPKD | 40  47  50 | Yes | *PKD1*  (AD; Monoallelic) | Het. | Non-synonymous SNV | c.12465T>G  p.Phe4155Leu | 0 | VUS  PM2, PP3, PP4  (considered disease-causing) |
|  | F70 | 10237 | M | ADPKD | 45  55  N/A | Yes | *PKD1*  (AD; Monoallelic) | Het. | Non-synonymous SNV | c.12386T>G  p.Met4129Arg | 0 | VUS  PM2, PP3, PP4 (considered disease-causing) |
|  | F613 | 8166 | M | PKD | 41  N/A  N/A | No | *PKD1*  (AD; Monoallelic) | Het. | Non-synonymous SNV | c.974A>G  p.Tyr325Cys | 0 | VUS  PM2, PP3, PP4, PP5 (considered disease-causing) |
|  | F642 | 8178 | M | PKD | 27  51  51 | No | *PKD1*  (AD; Monoallelic) | Het. | Non-synonymous SNV | c.9425T>C  p.Leu3142Pro | 0 | VUS  PM2,PP3, PP4, PP5 (considered disease-causing) |
|  | F374 | 10478 | M | ADPKD | 30  51  51 | Yes | *PKD1*  (AD; Monoallelic) | Het. | Non-synonymous SNV | c.6739G>C  p.Asp2247Pro | 0 | VUS  PM2, PP4, PP5 (considered disease-causing) |
|  | F317 | 10148 | F | ADPKD | 46  52  x | Yes | *PKHD1*  (AR; Biallelic) | Comp. Het. | Non-synonymous SNV | c.2702A>C  p.Asn901Thr | 0.00001989 | VUS  PM2, PP1, PP3 |
|  |  |  |  |  |  |  |  |  |  | c.107C>T  p.Thr36Met | 0.0005094 | Pathogenic  PS4, PM2, PM3, PP1, PP3, PP5 |
|  | F320 | 10173 | F | Normal size cystic  kidneys, intellectual impairment | 20  N/A  N/A | Yes | *MAP2K2* (AD; Monoallelic) | Het. | Non-synonymous SNV | c.692G>T  p.Arg231Leu | 0 | Likely Pathogenic  PM2, PP3, PP1, PP2 |
|  | F320 | 8104 | M | Normal size cystic  kidneys, intellectual impairment | 50  N/A  N/A | Yes | *MAP2K2* (AD; Monoallelic) | Het. | Non-synonymous SNV | c.692G>T  p.Arg231Leu | 0 | Likely Pathogenic  PM2, PP3, PP1, PP2 |
|  | F187 | 10064 | F | Small kidneys with subcortical cysts | 38  41  x | Yes | *PKHD1*  (AR; Biallelic) | Hom. | Non-synonymous SNV | c.5221G>A  p.Val1741Met | 0.00002849 | Likely Pathogenic  PS1, PM3, PP2, PP3, PP4 |
|  | F187 | 10262 | M | Small kidneys with subcortical cysts | 8  40  40 | Yes | *PKHD1*  (AR; Biallelic) | Hom. | Non-synonymous SNV | c.5221G>A  p.Val1741Met | 0.00002849 | Likely PathogenicPS1, PM3, PP2, PP3, PP4 |

*A priori* diagnosis, the clinical diagnosis of chronic kidney disease defined as per clinical and histological findings of the nephrologists’ referrals; A, adenine; ACMG, American College of Human Genetics Standards and Guidelines Classification; AD, autosomal dominant; ADTKD, autosomal dominant tubulointerstitial kidney disease; AR, autosomal recessive; AS/FSGS, Alport syndrome/focal segmental glomerulosclerosis; ASy, asymptomatic; Bx, biopsy; C, cytosine; c. change, nucleotide change; CAKUT, congenital anomalies of the kidney and urinary tract; CKD, chronic kidney disease; Comp., compound; Dx, diagnosis; ESKD, end stage kidney disease; ESS, essential splice site; F, female; FHx, documented family history of CKD; Fam ID, unique family identifier; fs, frameshift; GN, glomerulonephritis; G, guanine; Hemi., hemizygous; Het., heterozygous; Hom., homozygous; ID, unique individual identifier; ins; insertion; KTx, kidney transplantation M, male; MAF, minor allelic frequency; MPGN, membranoproliferative GN; N/A, not available; No., number; NPHP, nephronophthisis; PKD, polycystic kidney disease; p. change, amino acid change; SNV, single-nucleotide variant; T, thymine; TBM, thin basement membrane; TIKD, tubulointerstitial kidney disease; VUR, vesico-ureteric reflux; uCKD, chronic kidney disease of uncertain aetiology; XL, X-linked; x, not reached

^1^ gnomAD, variant frequencies listed for homozygous/ hemizygous (if applicable)/ heterozygous/ total alleles(http://gnomad.broadinstitute.org/)

^2^ in addition to three donors.

**Supplementary Table S2 ǀ** **Univariable analysis of factors favouring diagnostic outcome**

| **Variable** | **Odds ratio (95% conf. interval)** | ***P* value** |
| --- | --- | --- |
| Age | 1.01 (1.001 - 1.02) | 0.049 |
| Sex | 0.7 (0.5 - 1.03) | 0.085 |
| FHx of CKD | 4.4 (3 - 6.7) | <0.001 |
| Age of disease onset (<18 years) | 1.5 (0.9 - 2.04) | 0.004 |
| The underlying *a priori* diagnosis* | | |
| GN | 1 (referent) | |
| PKD | 18.9 (10.1 - 33.4) | <0.001 |
| TIKD | 6.9 (3.5 - 13.3) | <0.001 |
| AS | 26.5 (8.5 - 83) | <0.001 |
| FSGS | 9.3 (4 - 21.4) | <0.001 |
| uCKD | 14.6 (7.6 – 28.2) | <0.001 |
| CAKUT | 0.6 (0.2 - 1.2) | 0.191 |

AS, Alport syndrome; CAKUT, congenital anomalies of the kidney and urinary tract; ESKD, end stage kidney disease; FHx, family history; FSGS, focal segmental glomerulosclerosis; GN, glomerulonephritis; PKD, polycystic kidney disease; uCKD, CKD of uncertain aetiology; TIKD, tubulointerstitial kidney disease

**Supplementary Table S3 ǀ** **Multivariable** **analysis of factors favouring diagnostic outcome**

| **Variable** | **Odds ratio (95% conf. interval)** | ***P* value** |
| --- | --- | --- |
| Age | 0.99 (0.97 – 1.005) | 0.246 |
| Sex | 0.86 (0.58 – 1.28) | 0.471 |
| FHx of CKD | 3.69 (2.1 – 6.48) | <0.001 |
| Age of disease onset (<18 years) | 1.21 (0.79 – 1.85) | 0.376 |
| The underlying *a priori* diagnosis | | |
| GN | 1 (referent) | |
| PKD | 14.9 (8.04 – 27.61) | <0.001 |
| TIKD | 4.7 (2.15 – 10.3) | <0.001 |
| AS | 24.6 (6.4 – 93.1) | <0.001 |
| FSGS | 6.3 (2.3 – 17.4) | <0.001 |
| uCKD | 7.6 (1.7 – 32.4) | 0.006 |
| CAKUT | 0.6 (0.2 - 1.4) | 0.260 |

AS, Alport syndrome; CAKUT, congenital anomalies of the kidney and urinary tract; ESKD, end stage kidney disease; FHx, family history; FSGS, focal segmental glomerulosclerosis; GN, glomerulonephritis; PKD, polycystic kidney disease; uCKD, CKD of uncertain aetiology; TIKD, tubulointerstitial kidney disease


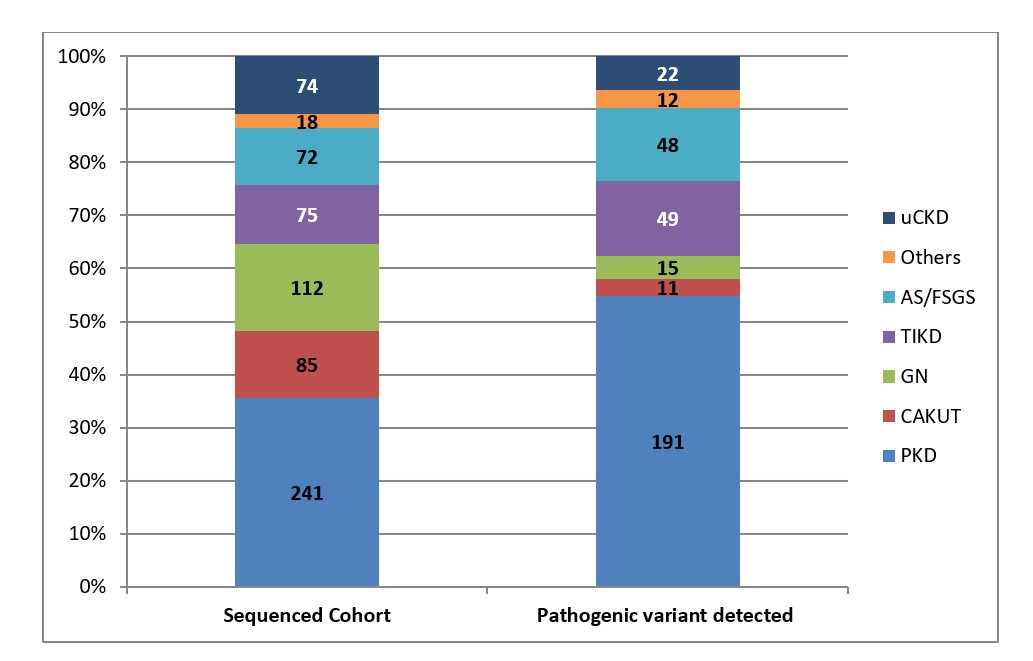
**Figure S1 ǀ Participants sequenced and solved in the Irish Kidney Gene Project (IKGP) broken down by *a priori* clinical diagnosis.** CAKUT, congenital anomalies of the kidney and urinary tract; FSGS, focal segmental glomerulosclerosis; GN, glomerulonephritis; PKD, polycystic kidney disease; uCKD, chronic kidney disease of uncertain aetiology; TIKD, tubulointerstitial kidney disease
